# The DSF Quorum Sensing System Controls the Positive Influence of *Stenotrophomonas maltophilia* on Plants

**DOI:** 10.1371/journal.pone.0067103

**Published:** 2013-07-18

**Authors:** Peyman Alavi, Henry Müller, Massimiliano Cardinale, Christin Zachow, María B. Sánchez, José Luis Martínez, Gabriele Berg

**Affiliations:** 1 Graz University of Technology, Institute of Environmental Biotechnology, Graz, Austria; 2 Austrian Centre of Industrial Biotechnology (ACIB GmbH), Graz, Austria; 3 Centro Nacional de Biotecnología (CNB-CSIC), Madrid, Spain; Wageningen University and Research Centre, Netherlands

## Abstract

The interaction of the Gram-negative bacterium *Stenotrophomonas maltophilia* with eukaryotes can improve overall plant growth and health, but can also cause opportunistic infections in humans. While the quorum sensing molecule DSF (diffusible signal factor) is responsible for the regulation of phenotypes in pathogenic *Stenotrophomonas*, up until now, no beneficial effects were reported to be controlled by it. Our objective was to study the function of DSF in the plant growth promoting model strain *S. maltophilia* R551-3 using functional and transcriptomic analyses. For this purpose, we compared the wild-type strain with a mutant deficient in the *rpfF* (regulation of pathogenicity factors) gene that is essential for the synthesis of DSF. Oilseed rape seeds treated with the wild-type strain showed a statistically significant increase in germination rate compared with those treated with the *rpfF* mutant. Similarly, the wild-type strain exhibited better plant growth promotion and a greater efficiency in colonizing oilseed rape compared to the mutant strain. Moreover, only the wild-type was capable of forming structured cell aggregates both *in vitro* and in the rhizosphere, a characteristic mediated by DSF. Gene transcription analyses showed that numerous genes known to play a role in plant colonization (e.g. chemotaxis, cell motility, biofilm formation, multidrug efflux pumps) are controlled by the *rpf*/DSF system in *S. maltophilia*. In addition, we detected new potential functions of spermidine, primarily for both growth promotion and stress protection. Overall, our results showed a correspondence between the regulation of DSF and the positive interaction effect with the plant host.

## Introduction

*Stenotrophomonas maltophilia* (syn. *Pseudomonas* and *Xanthomonas maltophilia*) is a type species within Gammaproteobacteria [1]. Although the species was isolated from diverse environments, plants are one of its main reservoirs [2]. In Brassicaceae oilseed rape, for example, these bacteria dominate the plant microbiome [2], [3]. *S. maltophilia* can be both seed-borne and occur with an endophytic lifestyle [2], [4]. The species is characterized by an extremely high intra-species diversity on the physiological and molecular level, especially within the environmental populations [1], [5]. Nevertheless, *S. maltophilia* strains are also nosocomial opportunistic pathogens. These clinical strains can cause disease with significant case/fatality ratios, especially in immunocompromised patients [6], [7]. Despite different approaches it was not possible to differentiate between environmental and clinical strains [8], [9], [10]. Interestingly, the mutation rate of *S. maltophilia* strains was the key to divide both groups. Clinical strains have a higher mutation rate than those from the environment and also contain hypermutators to help them quickly adapt once inside the fluctuating human body [11]. Sequence analysis of the first two known genomes of *S. maltophilia* is in favor of this assumption: the gene homology between *S. maltophilia* R551-3 and its clinical counterpart *S. maltophilia* K279a is approximately 85% [6]. While *S. maltophilia* belongs to the group of growth promoting rhizobacteria with biocontrol activity [12], [13], little is known about the mode of beneficial plant-microbe interactions [14]. Many strains are distinguished by unique mode of actions. Several strains are able to produce the phytohormone indole-3-acetic acid [15], while other *S. maltophilia* strains are free-living nitrogen-fixing bacteria [16]. Others can produce antifungal antibiotics [17], [18] or bioactive volatiles [19]. However, there is no past or current research concerning the regulation of these metabolites.

Quorum sensing systems based on N-acyl derivatives of homoserine lactones (N-AHLs) are often responsible for the regulation of various phenotypic characteristics in numerous plant-associated bacteria [20], [21]. AHL-based quorum sensing was not detected in *Stenotrophomonas*, but a diffusible signal factor (DSF)-based system, a novel quorum sensing system used by numerous xanthomonads [22], has been identified in clinical *S. maltophilia* K279a. In addition, structurally related systems have been detected in *Pseudomonas aeruginosa* and *Burkholderia cenocepacia* as well [23], [24]. DSF is a quorum sensing molecule of fatty acid nature that was first detected in *Xanthomonas* [25]. The *rpf* (regulation of pathogenicity factors) gene cluster is responsible for the synthesis and perception of DSF [25], and the *rpfF* gene product, known as DSF synthase, is essential for the synthesis of DSF in all known bacteria with this *rpf*/DSF system [23]. Each of the other members of the *rpf* gene locus (*rpfC*, *rpfG* and *rpfB*) fulfills a particular function with the RpfC/RpfG two-component system responsible for DSF perception and signal transduction [25], [26]. The *rpf*/DSF mechanism regulates a number of virulence-associated characteristics such as synthesis of extracellular enzymes, extracellular polysaccharides, and biofilm formation in various pathogenic strains [25], [26], [27]. While thoroughly studied in pathogenic species, especially in pathogenic xanthomonads, the *rpf*/DSF system is still unexamined in beneficial plant-associated bacteria.

The objective of this study was to investigate the role of DSF in the beneficial plant-associated *S. maltophilia* R551-3 model strain, originally isolated from the endosphere of poplar [14]. To address this question, we generated a DSF signal deficient mutant strain and investigated the role of the *rpf*/DSF signaling system with respect to bacteria-plant interactions by comparing the *S. maltophilia* R551-3 wild-type to the *rpfF* mutant strain.

## Materials and Methods

### Bacterial isolates and growth conditions

*Stenotrophomonas maltophilia* R551-3 was originally isolated from the endosphere of poplar [14]. Unless otherwise stated, the *S. maltophilia* R551-3 wild-type and *rpfF* mutant strains were cultivated at 37°C in Luria Bertani broth (Carl Roth, Germany). Overnight cultures were obtained by incubating the bacterial strains in LB medium at 37°C and 120 rpm for 18–20 h.

### Generation of the *S. maltophilia* R551-3 *rpfF* mutant strain

Generation of the *S. maltophilia* R551-3 *rpfF* mutant strain was performed according to Hoang et al. [28]. The *S. maltophilia* R551-3 *rpfF* gene sequence and its flanking regions were obtained from the genome database and specific primers were designed, as described below, to allow and confirm the generation of the *rpfF* knock-out strain. A DNA construct of 861 nucleotides consisting of the upstream and downstream flanking regions with respect to the *rpfF* gene in *S. maltophilia* R551-3 genome (NCBI Accession Nr.:CP001111) was obtained over a two-step PCR. In the first PCR, two amplicons were generated separately using primers R551-Up-F (cggaattcCAACCCGATTGCTGGAAGTAT) and R551-Up-R (cccctcactcctctccgtGACCAGTGCATTCCTGCC) which delivered the upstream flanking region of approximately 400 bp. Also, primers R551-Down-F (ggcaggaatgcactggtcACGGAGAGGAGTGAGGGG) and R551-Down-R (cccaagcttGCTTCAACGTGTACCCGAAC) were used to deliver the downstream flanking region of approximately 450 bp. The second PCR step was performed using primers R551-Up-F and R551-Down-R, delivering the desired gene construct, referred to as fragment C to maintain lucidity, which was subsequently cut with restriction endonucleases *Eco*RI and *Hin*dIII and ligated into the suicide vector pEX18Tc [28].

The suicide vector pEX18Tc possesses the tetracycline selectable (*tet*) marker and *sacB*, the sucrose counter-selectable marker. The vector was transferred into *S. maltophilia* R551-3 cells using tripartite mating [29]. The first and second crossover events were selected using tetracycline [20 µg ml^−1^] and 10% [w/v] sucrose, respectively. Generation of the *rpfF* mutant strain was confirmed, as described below, by performing separate PCRs, each with particular primers designed to confirm the recombination event and the generation of the *S. maltophilia* R551-3 knock-out *rpfF* mutant strain.

The primer pairs used for this purpose include R551-Up-F/R551-Down-R that would deliver a fragment consisting of the upstream and downstream regions and the *rpfF* gene in the *S. maltophilia* R551-3 wild-type strain while the mutant strain would solely deliver fragment C. Similarly, primers Deletion Check –F (CCAGGTTGCTGCCTCCAGCG) and Deletion Check –R (GTGATACGCCCGCCCCGTAAG) would only deliver a ca. 300 nucleotide-long inner part of the desired fragment C in the mutant strain while the wild-type would yield a fragment consisting of the *rpfF* gene and shorter-than-original flanking upstream and downstream regions attached to it. Primers R551-RpfF-F (GGTCGAACAGCACCTCCGGC) and R551-RpfF-R (ATCATCACCCGCCCCTCGCT) were specific for the *rpfF* gene and would deliver the product only in the wild-type. Moreover, primers Tet1 (AGCTGTCCCTGATGGTCGTC)/Tet2 (GAGCCTTCAACCCAGTCAGC) were used to confirm the elimination of the pEX18Tc suicide vector after the recombination event. All PCR results were sequenced in addition to confirmation on electrophoresis gel. Moreover to study the possible impact of the deletion of *rpfF* on the general growth of *S. maltophilia* R551-3, cultures of the wild-type and *rpfF* mutant strain were grown in liquid LB under 120 rpm at 37°C, and growth was assessed up to the mid-stationary phase. The deletion of the *rpfF* gene showed no impact on the growth rate of *S. maltophilia* R551-3.

### Confirmation of the incapability of the *S. maltophilia* R551-3 *rpfF* strain to produce DSF using glucanase plate assay

The incapability of the *S. maltophilia* R551-3 *rpfF*-deficient strain to produce DSF was confirmed using an assay based on the finding by Barber et al. [25] that showed the glucanase synthesis by *X. campestris* pv. *campestris* 8004 is DSF-dependent. The assay was performed on Petri dishes containing tryptone soy agar (TSA) (Carl Roth, Germany) supplemented with 1 g L^−1^ AZCL-Barley Beta-Glucan (Megazyme, Ireland). 1.5-cm-long streaks of the *Xanthomonas campestris* pv. *campestris rpfF* mutant strain were put on the plates, and supplemented with either sterile water (control), 100 µM cis-Δ2-11-methyl-Dodecenoic acid (Cayman Chemical Company, MI, USA) as synthetic DSF, supernatant extracts of a *S. maltophilia* R551-3 *rpfF*-deficient culture or supernatant extracts of a *S. maltophilia* R551-3 wild-type culture. The plates were incubated at 30°C for approximately 22 h and observed for glucanase production through formation of blue zones around the streaks.

To prepare supernatant extracts used in the glucanase assay from *S. maltophilia* R551-3 wild-type and *rpfF* mutant, 50 ml of 24-hour-incubation cultures were centrifuged at 10,000× *g* for 10 min. The supernatant was extracted twice with 1/5 vol. ethyl acetate (Carl Roth, Germany). For efficient phase separation, the samples were centrifuged at 7,500× *g* for 15 min. The ethyl acetate extract was completely evaporated and dissolved in 200 µl of sterile deionized water.

### RNA extraction and transcriptomic analyses

Quantitative sequencing of mRNA was used to assess gene expression. This characterization of bacterial transcriptomes based on ssRNA-seq is a novel and effective approach described by Perkins et al. [30]. To extract RNA, cultures of *S. maltophilia* R551-3 wild-type and *rpfF* mutant strains were grown in LB at 37°C and 120 rpm until the early stationary phase was reached, as the DSF concentration has been revealed to be highest at this point [25]. Cells were harvested using centrifugation at 5000× *g* for 3 min. RNA was extracted with the RNAprotect® Bacteria Reagent (Qiagen, Hilden, Germany), and rRNA was removed with MICROBExpress Kit (Invitrogen, Carlsbad, USA). The enriched mRNA was sequenced by LGC Genomics (Berlin, Germany) and data collections were performed by MicroDiscovery (Berlin, Germany).

### *Plant* experiments

Oilseed rape (cv. Californium, Kwizda, Austria) was used for plant assays. Prior to bacterial inoculation, seeds were surface sterilized using the seed infiltration approach described by Müller and Berg [31]. Seeds (0.7 g) were treated with 3% sodium hypochlorite (NaOCl) solution for 5 min and subsequently washed three times with sterile water. For inoculation, surface sterilized seeds were placed in a Petri dish and incubated in 10 ml of 0.85% NaCl solution containing 10^6^ CFU ml^−1^ of the bacterial culture on an orbital shaker at 85 rpm at room temperature for 3.5 hours. Colony forming units (CFU) ml^−1^ had been determined previously by diluting and plating 100 µl aliquots of 18-hour-old overnight cultures of the bacterial strains on LB plates. Colonies were counted after incubating the plates at 37°C for 48 h.

The impact of *S. maltophilia* R551-3′s on seed germination, plant growth, and the colonization of rhizosphere (root system) and aboveground plant parts was studied. To this end, two gnotobiotic systems were applied: seed germination pouches (mega international, MN, USA) and gnotobiotic soil. For microscopy, plant experiments were performed in seed germination pouches. In the approach using pouches, sterile pouches were loaded with inoculated seeds (5 each pouch) and moistened with 20 ml of sterile deionized water. The control group consisted of seeds incubated with 0.85% NaCl without bacteria. To avoid dehydration, the pouches were placed in sterile, covered polypropylene containers and incubated in a greenhouse at 23±2°C under artificial lighting (16 h light period) for 11 days. In the approach using the gnotobiotic soil system, polypropylene containers (4 L) were filled with 1.5 L of standard propagation soil (Empfinger Rindenmulch, Austria) and autoclaved to reduce the amount of soil-borne microorganisms and make possible the subsequent re-isolation of the strains. To test sterility, around 1 g of soil was suspended in 5 ml of sterile 0.85% NaCl solution and aliquots thereof were plated on LB Petri dishes which revealed that although significantly reduced in both the diversity and number of soil-borne microorganisms compared to non-autoclaved soil, the autoclaved soil was not absolutely sterile. Inoculated seeds were planted in the autoclaved soil (11 seeds pot^−1^) and watered with sterile water. The control group consisted of seeds incubated with 0.85% NaCl without bacteria. The plastic beakers were covered with sterile transparent lids and incubated in a greenhouse at 23±2°C under artificial lighting (16 h light period).

Bacterial colonization was assessed after 20 days. *S. maltophilia* wild-type and *rpfF* mutant strains were re-isolated from the 20-day-old oilseed rape plants using plant sections (roots with adhering soil, stem or leaves) sampled in sterile Whirlpack® bags (Carl Roth, Germany), supplemented with 5 ml 0.85% NaCl solution, and rigorously disintegrated using pestle and mortar. Subsequently, serial dilutions of the extract were plated onto LB plates. After incubation for 48 h at 37°C, the number of colonies was counted and the CFU g^−1^ plant material was assessed. The impact of the *S. maltophilia* R551-3 wild-type and *rpfF* mutant strains on seed germination was checked for as of the next day after sowing. The plant growth promoting effect was studied by measuring the fresh weight [g] of oilseed rape plants twice, after 5 and 20 days, respectively.

### GFP labeling of *S. maltophilia* R551-3 wild-type and *rpfF* strains

To study the capability of *S. maltophilia* R551-3 wild-type and *rpfF* mutant strains to form biofilm, the GFP-expressing plasmid pSM1890 [32] was introduced into the strains using tripartite mating as described by De Lorenzo and Timmis [29]. *gfp*-labeled cultures were grown in LB at 37°C and 120 rpm to OD_600_ of about 1, and then transferred into chambers purchased from Lab-Tek® II CC2™ Chamber Slide™ System (Thermo Fisher Scientific, NY, USA). These static cultures were then incubated at 37°C for three days. To remove the unbound bacteria, the medium was discarded and the glass slides were washed three times. This procedure was repeated four times. To complement the *rpfF* mutant strain, the culture medium was supplemented with 100 µM of synthetic DSF (cis-Δ2-11-methyl-Dodecenoic Acid, Cayman Chemical Company, MI, USA). Microscopic images were captured using a Leica TCS SPE confocal laser scanning microscope (Leica Microsystems, Wetzlar, Germany) with the Leica ACS APO 63X OIL CS objective (NA: 1.30). A z-step of 0.4–0.9 µm was applied to acquire confocal stacks. Photomultiplier gain and offset were individually optimized to improve the signal/noise ratio, and the 3D analysis of the stacks generated by confocal laser scanning microscopy (CLSM) was performed using Imaris 7.0 software (Bitplane, Zurich, Switzerland).

### Fluorescent *in situ* hybridization (FISH)

To study the ability of *S. maltophilia* R551-3 wild-type and *rpfF* mutant strains to colonize oilseed rape plants, FISH in combination with CLSM was used. To this end, oilseed rape roots which were colonized with the bacterial strains and grown in seed germination pouches were fixed with 4% paraformaldehyde/phosphate buffered saline (PBS) (3∶1 vol/vol). The control group contained roots with no bacterial treatment grown in the seed germination pouches. The fixed samples were then stored in PBS/96% ethanol (1∶1) at −20°C. The FISH probes were purchased from genXpress® (Wiener Neudorf, Austria), and the in-tube FISH was performed as described by Cardinale et al. [33]. The FISH probes used for the hybridization step were labeled with the fluorescent dye Cy3 and included EUB338 [34], EUB338 II, and EUB338 III [35], all directing eubacteria. An equimolar ratio of the FISH probes was used for the hybridization step to detect *S. maltophilia* wild-type and *rpfF* mutant strains. In this step, 15% formamide was added to the samples which were then subsequently incubated in a water bath (46°C) for 90 min. After hybridization, the samples were washed at 42°C for 15 min. Microscopy and image capturing were performed using a Leica TCS SPE confocal microscope (Leica Microsystems, Wetzlar, Germany) with the Leica ACS APO 63X OIL CS objective (NA: 1.30). A z-step of 0.2–0.9 µm was applied to acquire confocal stacks.

## Results

### Characterization of the *S. maltophilia* R551-3 *rpfF* mutant using glucanase plate assay and transcriptomic studies

In a first step, the *S. maltophilia* R551-3 *rpfF* mutant was characterized using the glucanase assay which proved that the *S. maltophilia* R551-3 *rpfF* mutant strain is incapable of producing DSF. The *X. campestris rpfF* mutant strain, when supplemented with either supernatant extracts from the *S. maltophilia* R551-3 wild-type culture or 100 µM synthetic DSF (cis-Δ2-11-methyl-dodecenoic acid), formed a blue zone due to the restored glucanase activity that leads to the degradation of the AZCL-Barley beta-glucan (Fig. S1). Conversely, no glucanase activity was observed after supplementing the *Xanthomonas rpfF* mutant with supernatant extracts from the *S. maltophilia* R551-3 *rpfF* mutant strain. Furthermore, transcriptomic analyses proved that the *rpfF* gene was transcribed only in the *S. maltophilia* R551-3 wild-type strain, as no *rpfF* mRNA could be detected for the *S. maltophilia* R551-3 *rpfF* mutant strain (locus tag: Smal_1830, Table S1).

### The effect of the *rpf*/DSF system on seed germination and plant growth promotion in *S. maltophilia* R551-3

**Figure 1:**
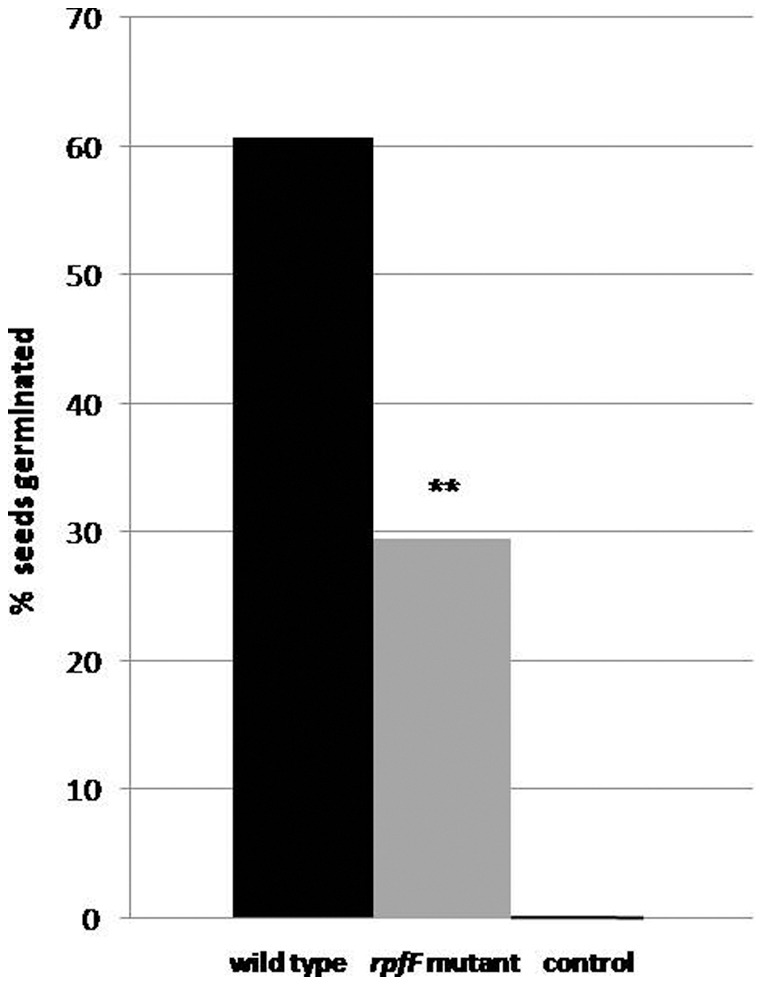
The role of the *S. maltophilia* R551-3 *rpf*/DSF system in seed germination. Bio-primed oilseed rape seeds were treated with 10^6^ CFU ml^−1^ *S. maltophilia* R551-3 wild-type or the *rpfF* mutant strain. The control plants were not treated with either of the bacterial strains, and showed no germination at all after 24 h of incubation. Oil seed rape seeds were planted into autoclaved soil and incubated under greenhouse conditions. The seed germination data represented here was obtained after 24 h of incubation. Data are presented as the mean values of germinated seeds of eight independent replicates. Each replicate consists of eleven surface sterilized, bio-primed oilseed rape seeds planted into soil. There was no seed germination for the control group after 24 h of incubation. **: p<0.05.

Oilseed rape seeds were inoculated with *Stenotrophomonas* (wild-type and *rpfF* mutant), planted into soil, and grown under greenhouse conditions. The number of germinated seeds was then compared one day after sowing (Fig. 1). The germination rate of the oilseed rape seeds treated with *S. maltophilia* R551-3 wild-type was twice that of seeds incubated with the *rpfF* mutant strain. Seeds which had not been inoculated with either of the strains (control seeds) showed no germination after 24 hours of incubation (Fig. 1).

However, the effect of the *rpf*/DSF system was confined to seed germination and, as described below, the early phase of plant growth. After five days of incubation in a greenhouse, the young oilseed plants were cut from the point of contact with the soil and weighed. The mean weight of 5-day-old plants inoculated with the wild-type strain was 0.154(±0.012) g, which is notably higher than those treated with the *rpfF* mutant (0.100[±0.014] g) and the non-inoculated control group (0.08[±0.01] g). At the end of the 20-day period, the plant growth promotion effect was less pronounced with no statistically significant difference (wild-type, 3.85[±0.67] g, the *rpfF* mutant strain, 3.49[±0.35] g and control, 3.06[±0.35] g). Moreover, the impact of *S. maltophilia* R551-3 on seed germination and plant growth was confined to the gnotobiotic soil system, as no effect was observed in the approach using sterile germination pouches.

### The effect of the *rpf*/DSF system on the plant colonization ability of *S. maltophilia* R551-3

**Figure 2:**
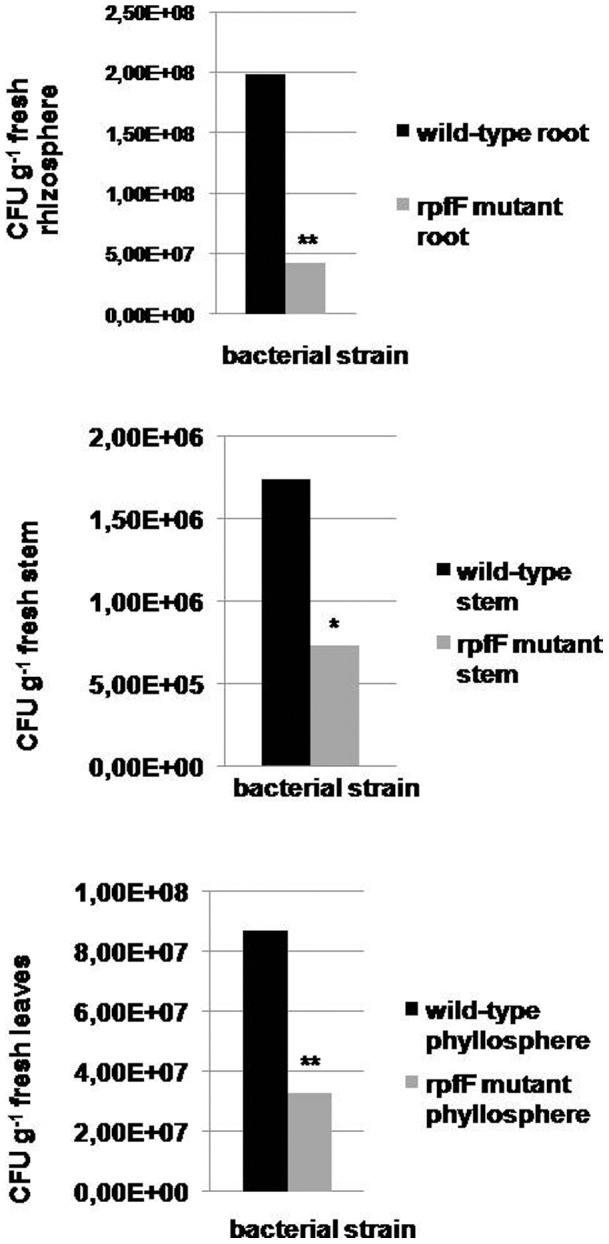
Colonization of oilseed rape plants by *S. maltophilia* R551-3 wild-type and *rpfF* mutant strains. Bacteria were re-isolated from 20-day-old oilseed rape plants grown in gnotobiotic soil systems under greenhouse conditions. For re-isolation, plant sections (roots with adhering soil, stem or leaves) were supplemented with 0.85% NaCl solution and rigorously disintegrated with pestle and mortar. Serial dilutions of the extract were then plated onto LB plates. After incubation at 37°C for 48 h, cell counts were determined and CFU g^−1^.

Oilseed rape plants were treated with *Stenotrophomonas* cells using seed-priming and cultivated in the gnotobiotic soil systems for 20 days to investigate the plant colonization efficiency of *S. maltophilia* R551-3 and to discern the possible role of the *rpf*/DSF system in plant colonization. Prior to sowing, the cell density attached to and within the seeds was evaluated. The seed inoculation was similar for both strains and resulted in 1.44×10^6^(±1.55×10^5^) and 1.48×10^6^(±7.8×10^4^) CFU seed ^−1^ for the wild-type and *rpfF* mutant strain, respectively. At the end of the incubation period, the plant sections (rhizosphere, stems, leaves) were dissected and *Stenotrophomonas* cells were subsequently re-isolated. In general, both strains were capable of colonizing the rhizosphere and phyllosphere (stem and leaves), however the *S. maltophilia* R551-3 wild-type strain showed a significantly higher colonization of all parts of the oilseed rape plant in comparison with the *rpfF* mutant (Fig. 2). In addition to the soil system, plants were grown in sterile seed germination pouches. The cell count obtained from the stem of plants inoculated with wild-type was higher (7.8×10^7^[±2.7×10^7^] CFU g^−1^) than that of the *rpfF* mutant strain (3.4×10^7^[±1.3×10^7^] CFU g^−1^), as well as from the leaves of the oilseed rape plants, respectively, (9.8×10^7^[±2.6×10^7^] CFU g^−1^ and 4.8×10^7^[±2.0×10^7^] CFU g^−1^).

**Figure 3:**
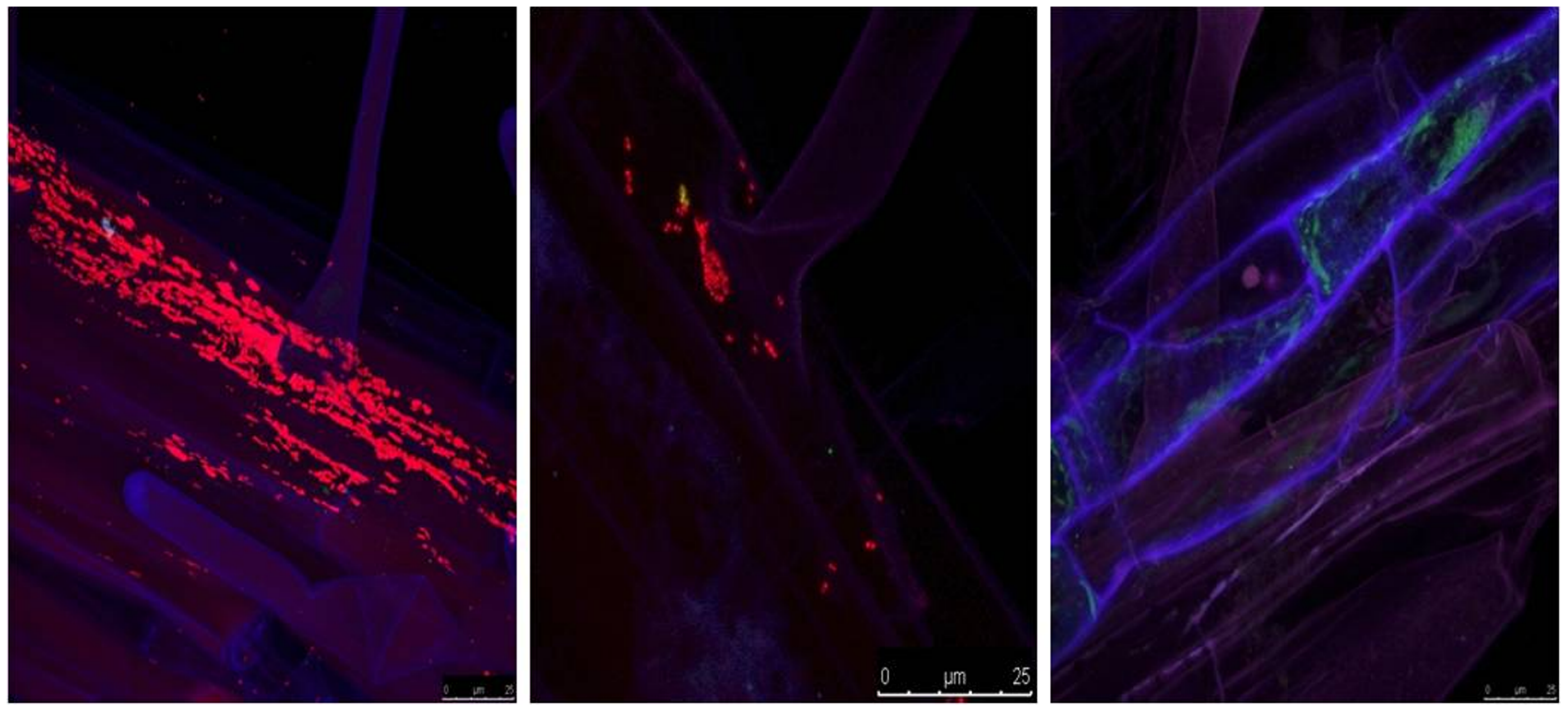
Colonization of the 11-day-old oilseed rape rhizosphere by the wild-type (left) and the *rpfF* mutant strain (middle) visualized by fluorescent *in situ* hybridization (FISH). The image on the right-hand side corresponds to the seeds without bacterial inoculation (control). An equimolar ratio of the FISH probes EUB338, EUB338 II and EUB338 III labeled with the fluorescent dye Cy3 was used in the hybridization step for the detection of *S. maltophilia* wild-type and *rpfF* mutant strains. Microscopic images were captured using a Leica TCS SPE confocal microscope. The Leica ACS APO 63X OIL CS objective (NA: 1.30) was used to acquire confocal stacks by applying a z-step of 0.2–0.9 µm. Same colonization pattern was obtained for at least four samples from separate replicates.

In addition, the colonization of the oilseed rape rhizosphere by *S. maltophilia* R551-3 wild-type and the *rpfF* mutant strain was also investigated using FISH combined with CLSM (Fig. 3). We found similar results to those achieved in re-isolation assays in which the wild-type strain colonized oilseed rape more intensely than the *rpfF* mutant strain (Fig. 3). In addition, we observed a compact organization of the wild-type cells in the oilseed rape rhizosphere. The *rpfF* mutant strain, however, sparsely colonized the rhizosphere with bacterial cells scattered throughout the oilseed rape root (Fig. 3).

### The impact of DSF on the formation of biofilm-like structures

**Figure 4:**
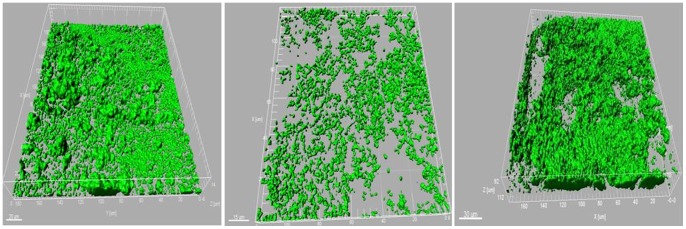
3D CLSM images were captured from the *gfp*-labeled *S. maltophilia* wild-type (left), *rpfF* mutant strain (middle) and the *rpfF* mutant strain supplemented with 100 µM DSF (right). While the wild-type strain formed structured, surface-covering cell architecture with a particular texture consisting of several cell layers, the *rpfF* mutant strain constructed an unstructured and unconnected monolayer film of cells. The *rpfF* mutant strain supplemented with 100 µM DSF (cis-Δ2-11-methyl-Dodecenoic Acid), however, formed the same structure observed for the wild-type. *gfp*-labeled wild-type and *rpfF* mutant strain cultures as well as the *rpfF* mutant strain culture supplemented with 100 µM synthetic DSF molecule were grown in LB medium up to OD_600_ of 1. The cultures were then filled into the chambers of the Lab-Tek® II CC2™ Chamber Slide™ System and incubated at 37°C for three days. To capture the microscopic images a Leica TCS SPE confocal laser scanning microscope was used. The confocal stacks were acquired with the Leica ACS APO 63X OIL CS objective (NA: 1.30) by applying a z-step of 0.4–0.9 µm. The 3D analysis of the CLSM stacks was performed using the software Imaris 7.0. The assay was performed at least four times.

In order to investigate the possible role of the *rpf*/DSF system in *S. maltophilia* R551-3, *gfp*-labeled wild-type and *rpfF* mutant strains were generated and cultivated in glass chambers. The CLSM and 3D analysis of the glass slides showed that the *S. maltophilia* R551-3 wild-type strain formed structured, surface-covering cell architecture with a particular texture consisting of several cell layers. Conversely, the *rpfF* mutant strain solely constructed an unstructured and unconnected monolayer film of cells. The *rpfF* mutant strain supplemented with 100 µM DSF (cis-Δ2-11-methyl-Dodecenoic Acid), however, constructed the same structure observed for the wild-type. Fig. 4 represents the 3D CLSM images captured from *gfp*-labeled wild-type, *rpfF* mutant, and the *rpfF* mutant strain supplemented with DSF.

### The impact of the *rpf*/DSF system on the genome expression of *S. maltophilia* R551-3

Comparing the transcriptomic data of the *S. maltophilia* R551-3 wild-type to the *rpfF* mutant strain revealed that the *rpf*/DSF system regulates the expression of numerous key genes that both directly and indirectly are involved in plant growth promotion and biocontrol including those coding for cell motility, chemotaxis, LPS structure, biofilm formation, iron transport, antibiotic resistance, and also stress resistance through the synthesis of chaperone proteins (Table 1). Of the total genes either up or down-regulated, only those showing fold changes greater than or equal to 1.5 and less than or equal to 0.5 were considered significantly impacted. The complete list of genes with a significant transcription fold change is provided in Table S1.

**Table 1:** Transcription ratio of the *S. maltophilia* R551-3 wild-type compared to the *rpfF* mutant strain for selected genes that code for products of important physiological role with regard to bacteria-plant interactions.

Physiological role	Gene/locus	Product/function	Fold change wild-type/*rpfF* mutant
**cell motility**	Smal_1869	Flagellar export protein FliQ	35.1
	fliN	flagellar motor switch protein FliN	28.2
	Smal_1868	flagellar biosynthetic protein	17.9
	Smal_1877	flagellar related ATPase	17.4
	Smal_1881	flagellar hook-basal body complex subunit	12.9
	motC	flagellar motor protein	9.9
	flgA	flagellar basal body P-ring biosynthesis protein	4.5
	Smal_1874	flagellar basal body-associated protein	4.5
	Smal_1863	cobyrinic acid ac-diamide synthase	3.2
	Smal_1894	flagellin	3.1
	flgK	flagellar hook-associated protein	3.1
	Smal_1909	putative anti-sigma-28 factor	2.4
	Smal_1876	flagellar export protein	2.3
	flgJ	flagellar rod assembly protein	2.1
**chemotaxis**	Smal_1907	response regulator receiver CheW	15.0
	Smal_1846	methyl-accepting chemotaxis sensor molecule	6.3
	Smal_1855	chemotaxis signal transducer	2.7
	Smal_1845	CheR-type chemotaxis methyltransferase	2.7
**direct plant growth promoting**	Smal_2304	putative spermidine export protein *	43.8
	Smal_2305	putative spermidine export protein *	5.4
	Smal_3769	spermidine synthase	1.5
	Smal_1341	spermidine/putrescine-binding periplasmic protein	1.5
**LPS biosynthesis**	Smal_0537	permease	2.1
	Smal_1400	3-deoxy-manno-octulosonate cytidylyltransferase	1.9
	Smal_3508	UDP-N-acetylglucosamine pyrophosphorylase	1.4
**biofilm formation**	Smal_2718	polysaccharide polymerase involved in biofilm formation	3.8
	Smal_0846	serine-threonine phosphatase	2.0
	Smal_2717	polysaccharide deacetylase	-5.5
**iron transport**	Smal_1803	iron transport protein	1.8
**antibiotic resistance and multidrug pumps**	Smal_2304	multidrug resistance protein	43.8
	Smal_2305	small multidrug resistance protein	5.4
	Smal_2146	beta-lactamase	2.3
	Smal_1568	multidrug efflux transporter (SmeW)	2.7
**chaperone synthesis**	Smal_1891	flagellin-specific chaperone	50.2
	Smal_1916	DnaJ class molecular chaperone	15.0
	Smal_1271	pili assembly chaperone	14.2
	Smal_1576	severe stress chaperone	0.2

Genes listed here play crucial roles in plant colonization, biofilm formation, ecological persistence, biocontrol and plant growth promotion. *: Originally annotated as multidrug resistance protein, but blastp analysis revealed significant homology to the mdtJ, the spermidine export protein of the plant growth promoting and biocontrol agent *S. r,hizophila* DSM14405^T^.

Regarding cell motility, genes coding for the flagellar machinery as well as chemotaxis in *S. maltophilia* R551-3 are strongly controlled by the *rpf*/DSF system, as they are positively regulated by DSF as shown in Table 1. Some of these genes, Smal_1868 (coding for flagellar biosynthetic protein; expression fold change of 17.9) and Smal_1869 (coding for flagellar export protein; expression fold change of 35.1) for instance, are significantly up-regulated by DSF. Regarding plant growth promotion, the *S. maltophilia* R551-3 *rpfF* mutant strain showed a slight decrease in the expression of the spermidine synthase gene, coding for a well-known growth regulator. In addition, the expression of two adjacent genes, Smal_2304 and Smal_2305, that code for spermidine export proteins is highly DSF-dependent, as the corresponding expression fold changes of 43.8 and 5.4, respectively suggest. As for surface adherence, genes that play a role in LPS biosynthesis and biofilm formation are positively regulated by the *rpf*/DSF system with the exception of Smal_2717, a polysaccharide deacetylase gene that affects biofilm formation, which shows a wild-type/*rpfF* mutant expression fold change of -5.5. Furthermore, genes involved in iron transport, antibiotic resistance, and those responsible for stress resistance through biosynthesis of chaperones are also positively regulated by DSF in *S. maltophilia* R551-3, as presented in Table 1.

## Discussion

*Stenotrophomonas maltophilia* is known for its ambiguous interaction with eukaryotic hosts. We found that the quorum sensing system DSF is involved in many beneficial interactions such as plant growth promotion as well as plant colonization in *S. maltophilia* R551-3. The plant growth promoting effect, however, is not a result of the regulation of indole-3-acetic acid (IAA) synthesis, extracellular proteases, or volatile organic compounds as the respective physiological tests showed no significant difference between the wild-type and *rpfF* mutant strains (data not shown). However, the transcriptomic analysis indicated that numerous genes crucial for bacteria-plant interactions are regulated by the *rpf*/DSF quorum sensing in *S. maltophilia* R551-3. With this new information, we can analyze the function of these DSF-dependent cellular mechanisms that underlie both plant colonization and growth promotion in *S. maltophilia*. However, it should be noted that although the transcriptomic analyses were very helpful in understanding the role of the *rpf*/DSF system in *S. maltophilia* R551-3, more investigations also under different conditions are needed to understand the whole functioning.

Flagella-dependent motility and chemotaxis are important factors in biofilm formation [36] and plant colonization [37]. As represented in Fig. 4, only *S. maltophilia* R551-3 wild-type formed the structured surface-covering and multi-layer biofilm-like cell architecture on glass slides. According to numerous studies, the *rpf*/DSF system has been shown to play an important role in forming cell aggregates, surface attachment, and surface adherence in various pathogenic xanthomonads [22], [35]. Furthermore, biofilm formation was found both negatively [38] and positively [39] regulated by the *rpf*/DSF system. Our findings on quorum sensing-dependent biofilm formation in the plant-associated *S. maltophilia* R551-3 are similar to those by Torres et al. [39]. Moreover, the transcriptomic analyses indicated that the expression of flagellar apparatuses as well as biofilm formation are controlled by the *rpf*/DSF system. Given these results from physiological and transcriptomic approaches the *rpf*/DSF quorum sensing system controls the genes responsible for biofilm formation and plant colonization, which in turn play an important role in the interaction between *S. maltophilia* R551-3 and plants.

*S. maltophilia* R551-3 promotes seed germination and plant growth, however it is controversial which mechanism(s) causes this positive interaction. Only low levels of the plant growth hormone indole-3-acetic acid (IAA) are produced by the bacterium, [14] and no difference was observed for IAA synthesis between the wild-type and the *rpfF* mutant strain. In addition, the *S. maltophilia* R551-3 genome does not contain typical plant growth promoting genes that are found in other plant-beneficial bacteria, such as genes for the metabolism of plant signal molecules (e.g.: γ-amino butyric acid and phenyl acetic acid) or those for the synthesis of acetoin [6]. Nevertheless, our transcriptomic analyses revealed that a gene with significant homology to spermidine synthase was down-regulated in the *S. maltophilia* R551-3 *rpfF* mutant strain (wild-type/mutant fold change of 1.5; Table 1). Spermidine is a well-known plant growth regulator and has been recently shown to strongly promote the growth of arugula plants [40]. Furthermore, spermidine affects biofilm formation in various bacterial species via multiple pathways that involve both transport and signaling networks [41]. In addition to spermidine synthase, two adjacent genes (Smal_2304 and Smal_2305) in the chromosome of *S. maltophilia* R551-3 are also strongly regulated by the *rpf*/DSF system, as they were down-regulated by 43.8 and 5.4 folds (Table 1) respectively in the *rpfF* mutant strain. Although originally annotated as multidrug-coding genes with unknown functions, the amino acid sequences of Smal_2304 and Smal_2305 showed significant similarity to the spermidine export protein of the plant growth promoting and biocontrol agent *S. rhizophila* DSM14405^T^. For this strain, besides glucosylglycerol spermidine production as well as export was found as main stress response against high salinities but also to oilseed rape root exudates [42]. In this way, a higher level of spermidine synthase combined with highly active spermidine export proteins regulated in *S. maltophilia* R551-3 could result in a notably higher spermidine concentration in oilseed rape seeds, thus leading to enhanced germination and growth promotion.

Biological control of pathogens can indirectly result in plant growth promotion [43]. According to the transcriptomic data and the finding that the *rpf*/DSF-dependent seed germination and plant growth promoting effects are present only in the gnotobiotic soil system, but not the sterile germination pouches, biocontrol can indirectly be involved in causing seed germination and plant growth promotion in oilseed rape by *S. maltophilia* R551-3. Numerous studies have focused on mechanisms underlying biocontrol [44], [45], [46], and according to the transcriptomic analyses, a number of these mechanisms that are indicated to be DSF-dependent in *S. maltophilia* R551-3, are briefly discussed here. For instance, competing over iron through its efficient transport is a biocontrol mechanism [46] that is positively regulated by the *rpf*/DSF system in *S. maltophilia* R551-3. Another important biocontrol mechanism is the ability to compete over nutrients and niches through plant colonization [44], [45]. In this regard, the *S. maltophilia* R551-3 *rpfF* mutant strain is impaired in root/plant colonization that could be driven by the down-regulation of the flagellar machinery. Other biocontrol mechanisms that are indicated to be DSF-dependent in *S. maltophilia* R551-3 include the biosynthesis of antibiotic (beta-lactamase) and multidrug efflux pumps.

In conclusion, this study demonstrated that the significance of the *rpf*/DSF quorum sensing system is not confined to virulence caused by pathogenic bacteria [23], [47], but also used by the plant-associated biocontrol agent *S. maltophilia* R551-3 that underlies its role in positive plant-microbe interactions. Furthermore, the dual role of quorum sensing systems as beneficial and harmful is of relevance for understanding the interactions of both opportunistic and beneficial bacteria with their hosts in hospitals and in the field.

## Supporting Information

Figure S1 **Physiological verification of the loss of DSF production by the** ***S. maltophilia*** **R551-3** ***rpfF*** **-deficient mutant strain using the DSF-dependent endoglucanase activity of** ***X. campestris*** **; The endoglucanase assay was carried out on Petri dishes containing tryptone soy agar (TSA) supplemented with 1 g L^−1^ AZCL-Barley Beta-Glucan.** The bacterial streaks, marked as red-colored lines for better illustration purposes, correspond to the *Xanthomonas campestris* pv. *campestris rpfF* mutant strain (Barber et al., 1997) grown on Petri dishes: A: *X. campestris* 8004 *rpfF*-deficient mutant strain supplemented with sterile water (control) B: *X. campestris* 8004 *rpfF*-deficient mutant supplemented with 100 µM synthetic DSF C: *X. campestris* 8004 *rpfF*-deficient mutant supplemented with supernatant extracts of a *S. maltophilia* R551-3 *rpfF*-deficient culture D: *X. campestris* 8004 *rpfF*-deficient mutant supplemented with supernatant extracts of a *S. maltophilia* R551-3 wild-type culture. The blue zone formed around the *X. campestris* streak in B and D corresponds to the degradation of AZCL-Barley Beta-Glucan due to the production of extracellular glucanases. Supplementing the *X. campestris* 8004 *rpfF*-deficient strain with both synthetic DSF and supernatant extracts from the *S. maltophilia* R551-3 wild-type culture restored its ability to produce extracellular glucanase. In contrast, the treatment of the *X. campestris* 8004 *rpfF*-deficient mutant strain with supernatant extracts of the *S. maltophilia* R551-3 *rpfF*-deficient mutant strain failed to restore the glucanase activity (C). Same results were obtained for a total of four replicates.
(TIF)

Table S1 **The complete list of the genes with a significant transcription fold change being regulated by DSF in** ***S. maltophilia*** **R551-3.**
(PDF)
