# Supplementary figures and images for: The DSF Quorum Sensing System Controls the Positive Influence of Stenotrophomonas maltophilia on Plants
Source: PLoS One. 2013 Jul 18;8(7):e67103. doi: 10.1371/journal.pone.0067103 (PMC3715506; doi:10.1371/journal.pone.0067103)

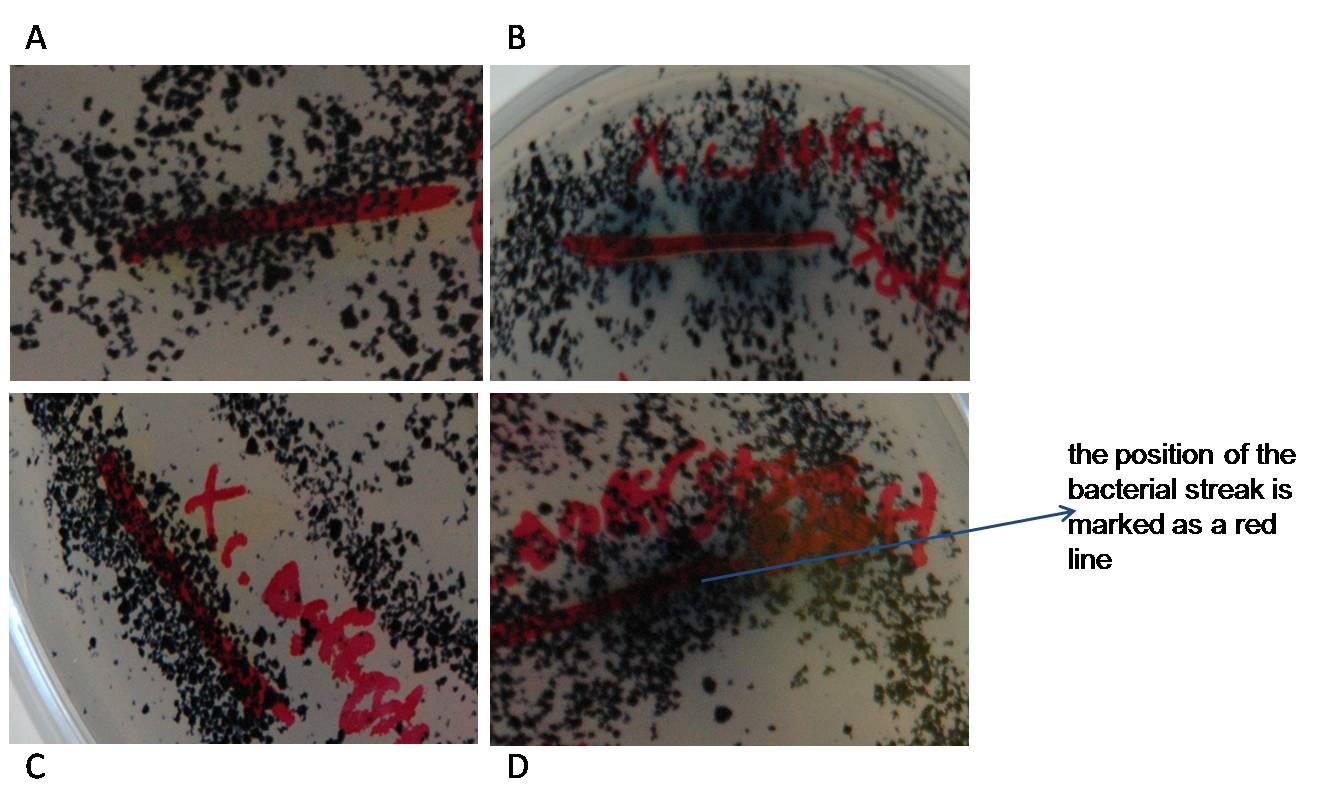

Supplement: Figure S1 — Physiological verification of the loss of DSF production by the S. maltophilia R551-3 rpfF -deficient mutant strain using the DSF-dependent endoglucanase activity of X. campestris ; The endoglucanase assay was carried out on Petri dishes containing tryptone soy agar (TSA) supplemented with 1 g L−1 AZCL-Barley Beta-Glucan. The bacterial streaks, marked as red-colored lines for better illustration purposes, correspond to the Xanthomonas campestris pv. campestris rpfF mutant strain (Barber et al., 1997) grown on Petri dishes: A: X. campestris 8004 rpfF-deficient mutant strain supplemented with sterile water (control) B: X. campestris 8004 rpfF-deficient mutant supplemented with 100 µM synthetic DSF C: X. campestris 8004 rpfF-deficient mutant supplemented with supernatant extracts of a S. maltophilia R551-3 rpfF-deficient culture D: X. campestris 8004 rpfF-deficient mutant supplemented with supernatant extracts of a S. maltophilia R551-3 wild-type culture. The blue zone formed around the X. campestris streak in B and D corresponds to the degradation of AZCL-Barley Beta-Glucan due to the production of extracellular glucanases. Supplementing the X. campestris 8004 rpfF-deficient strain with both synthetic DSF and supernatant extracts from the S. maltophilia R551-3 wild-type culture restored its ability to produce extracellular glucanase. In contrast, the treatment of the X. campestris 8004 rpfF-deficient mutant strain with supernatant extracts of the S. maltophilia R551-3 rpfF-deficient mutant strain failed to restore the glucanase activity (C). Same results were obtained for a total of four replicates. (TIF) [file pone.0067103.s001.tif]
