# Supplementary material for: The DSF Quorum Sensing System Controls the Positive Influence of Stenotrophomonas maltophilia on Plants
Source: PLoS One. 2013 Jul 18;8(7):e67103. doi: 10.1371/journal.pone.0067103 (PMC3715506; doi:10.1371/journal.pone.0067103)
Supplement: Table S1 — The complete list of the genes with a significant transcription fold change being regulated by DSF in S. maltophilia R551-3. (PDF) [file pone.0067103.s002.pdf]

Supplementary Table 1: The complete list of the genes with a significant transcription fold change being regulated by DSF in *S. maltophilia* R551-3

The minus sign before fold changes corresponds to down regulation of the corresponding genes in the *rpfF* mutant strain.

locus tag	fold change wild-type/*rpfF* mutant	(putative) product
Smal_0012	2.26	phospholipase D/transphosphatidylase
Smal_0013	3.62	hypothetical protein
Smal_0014	1.51	pyridoxine 5'-phosphate synthase
Smal_0018	4.52	hypothetical protein
Smal_0022	2.26	TonB-dependent receptor
Smal_0023	13.72	hypothetical protein
Smal_0025	0.36	Ku protein
Smal_0027	0.18	hypothetical protein
Smal_0030	0.30	cysteine dioxygenase type I
Smal_0031	1.56	LacI family transcriptional regulator
Smal_0036	1.51	hypothetical protein
Smal_0037	1.71	TonB-dependent receptor
Smal_0038	0.23	acid phosphatase
Smal_0040	-4.34	hypothetical protein
Smal_0043	42.65	hypothetical protein
Smal_0044	2.11	hypothetical protein
Smal_0045	2.71	formamidopyrimidine-DNA glycosylase
Smal_0047	2.20	thymidine kinase
Smal_0049	3.16	Sel1 domain-containing protein repeat-containing protein
Smal_0051	2.11	acetyltransferase
Smal_0052	1.81	hypothetical protein
Smal_0055	1.81	hypothetical protein
Smal_0056	1.57	glycerol-3-phosphate acyltransferase
Smal_0057	23.25	hypothetical protein
Smal_0060	2.71	hypothetical protein
Smal_0061	1.81	hypothetical protein
Smal_0066	-4.93	hypothetical protein
Smal_0067	3.62	hypothetical protein
Smal_0068	-9.44	short-chain dehydrogenase/reductase SDR
Smal_0069	0.45	hypothetical protein
Smal_0070	1.81	hypothetical protein
Smal_0071	0.30	beta-lactamase

Smal_0072	-11.02	AraC family transcriptional regulator
Smal_0074	1.81	major facilitator superfamily protein
Smal_0075	0.23	TonB-dependent receptor
Smal_0076	12.23	CopY family transcriptional repressor
Smal_0077	2.37	peptidase M56 BlaR1
Smal_0078	0.45	lipoprotein
Smal_0081	1.58	hypothetical protein
Smal_0082	2.71	hypothetical protein
Smal_0085	2.71	hypothetical protein
Smal_0090	2.36	peptidase U62 modulator of DNA gyrase
Smal_0092	30.75	hypothetical protein
Smal_0096	1.88	hypothetical protein
Smal_0097	0.45	ATPase AAA
Smal_0098	1.62	partition protein
Smal_0102	3.16	thioesterase superfamily protein
Smal_0104	6.33	hypothetical protein
Smal_0105	1.89	hypothetical protein
Smal_0110	0.34	nitrogen regulatory protein P-II
Smal_0111	1.66	ammonium transporter
Smal_0114	5.43	hypothetical protein
Smal_0116	1.81	LytTR family two component transcriptional regulator
Smal_0118	0.30	alpha/beta hydrolase fold domain-containing protein
Smal_0120	3.32	hypothetical protein
Smal_0124	2.55	copper/zinc binding superoxide dismutase
Smal_0129	1.66	HemY domain-containing protein
Smal_0131	0.30	uroporphyrinogen-III synthase
Smal_0134	1.91	rhodanese domain-containing protein
Smal_0146	1.81	Glyoxalase/bleomycin resistance protein/dioxygenase
Smal_0148	0.18	hypothetical protein
Smal_0155	22.20	two component LuxR family transcriptional regulator
Smal_0157	1.51	cytochrome c class I
Smal_0158	1.70	electron-transferring-flavoprotein dehydrogenase
Smal_0161	-82.34	carboxymuconolactone decarboxylase
Smal_0162	0.30	4-oxalocrotonate tautomerase
Smal_0164	19.83	hypothetical protein
Smal_0167	1.71	3-oxoacyl-ACP synthase
Smal_0168	1.63	alpha/beta hydrolase fold domain-containing protein
Smal_0169	-12.28	hypothetical protein

Smal_0178	2.11	hypothetical protein
Smal_0179	2.45	peptidyl-tRNA hydrolase domain-containing protein
Smal_0182	-11.70	3-dehydroquinate dehydratase
Smal_0183	11.77	hypothetical protein
Smal_0186	0.26	LysR family transcriptional regulator
Smal_0187	0.45	LysR family transcriptional regulator
Smal_0188	0.36	malate synthase
Smal_0190	2.26	cyclic nucleotide-binding protein
Smal_0195	0.28	hypothetical protein
Smal_0199	0.13	TetR family transcriptional regulator
Smal_0202	0.30	hypothetical protein
Smal_0203	2.35	hypothetical protein
Smal_0208	37.17	lysine exporter protein LysE/YggA
Smal_0210	0.30	NAD-dependent deacetylase
Smal_0212	0.45	LysR family transcriptional regulator
Smal_0214	0.18	nicotinamidase
Smal_0215	0.26	hypothetical protein
Smal_0217	3.62	hypothetical protein
Smal_0220	1.51	acyl-CoA dehydrogenase domain-containing protein
Smal_0222	1.54	enoyl-CoA hydratase/isomerase
Smal_0224	1.96	OsmC family protein
Smal_0228	1.61	hypothetical protein
Smal_0235	1.81	sporulation domain-containing protein
Smal_0236	5.43	addiction module killer protein
Smal_0237	-86.43	putative transcriptional regulator
Smal_0238	1.61	arginase
Smal_0239	0.36	entericidin EcnAB
Smal_0243	-51.86	hypothetical protein
Smal_0246	-59.15	hypothetical protein
Smal_0248	0.26	hypothetical protein
Smal_0249	2.03	RarD protein, DMT superfamily transporter
Smal_0251	0.45	hypothetical protein
Smal_0252	3.62	hypothetical protein
Smal_0253	0.23	ABC transporter-like protein
Smal_0254	11.30	RND family efflux transporter MFP subunit
Smal_0255	4.52	ABC transporter-like protein
Smal_0260	0.45	Catalase domain-containing protein
Smal_0265	2.41	hypothetical protein

Smal_0266	2.66	hypothetical protein
Smal_0270	0.30	hypothetical protein
Smal_0274	0.26	hypothetical protein
Smal_0281	6.71	DNA repair protein RadC
Smal_0283	-16.68	two component LuxR family transcriptional regulator
Smal_0288	0.30	osmosensitive K+ channel signal transduction histidine kinase
Smal_0289	1.81	potassium-transporting ATPase subunit C
Smal_0291	-15.27	potassium-transporting ATPase subunit A
Smal_0292	0.45	hypothetical protein
Smal_0293	2.51	hypothetical protein
Smal_0300	2.01	tyrosyl-tRNA synthetase
Smal_0303	1.64	peptidase M23
Smal_0304	1.60	carboxyl-terminal protease
Smal_0307	2.71	nicotinamide mononucleotide transporter PnuC
Smal_0311	1.55	cytochrome c oxidase subunit I
Smal_0312	40.52	hypothetical protein
Smal_0313	1.81	cytochrome C oxidase assembly protein
Smal_0314	1.58	cytochrome c oxidase subunit III
Smal_0315	0.45	hypothetical protein
Smal_0316	6.48	hypothetical protein
Smal_0321	1.81	bile acid:sodium symporter
Smal_0329	1.58	dihydroneopterin aldolase
Smal_0331	2.05	glycoside hydrolase family 3
Smal_0335	14.35	hypothetical protein
Smal_0337	2.71	hypothetical protein
Smal_0339	0.11	mandelate racemase/muconate lactonizing protein
Smal_0342	0.45	TonB-dependent receptor plug
Smal_0344	3.62	peptidase M15D vanX D-ala-D-ala dipeptidase
Smal_0353	2.71	hypothetical protein
Smal_0356	1.81	hypothetical protein
Smal_0362	17.00	hypothetical protein
Smal_0364	1.54	PadR-like family transcriptional regulator
Smal_0367	-56.74	hypothetical protein
Smal_0368	8.17	TonB family protein
Smal_0370	48.17	hypothetical protein
Smal_0372	3.44	hypothetical protein
Smal_0382	16.42	hypothetical protein
Smal_0386	0.45	hypothetical protein

Smal_0387	-12.91	alpha/beta hydrolase fold domain-containing protein
Smal_0389	3.01	hypothetical protein
Smal_0391	0.45	polysaccharide deacetylase
Smal_0392	6.33	hypothetical protein
Smal_0393	-15.77	hypothetical protein
Smal_0394	-25.10	hypothetical protein
Smal_0397	2.71	hypothetical protein
Smal_0398	1.81	hypothetical protein
Smal_0399	0.45	cobalamin synthesis protein P47K
Smal_0400	0.45	ABC transporter-like protein
Smal_0402	-4.43	beta-lactamase
Smal_0403	1.81	major facilitator superfamily protein
Smal_0404	0.45	EAL domain-containing protein
Smal_0410	11.43	DGPFAETKE family protein
Smal_0413	-14.66	hypothetical protein
Smal_0414	0.45	putative ECF subfamily RNA polymerase sigma-24 subunit
Smal_0420	0.45	signal transduction histidine kinase LytS
Smal_0421	-27.12	LytTR family two component transcriptional regulator
Smal_0422	13.72	hypothetical protein
Smal_0424	5.43	hypothetical protein
Smal_0425	8.04	TetR family transcriptional regulator
Smal_0426	0.45	major facilitator superfamily protein
Smal_0427	-26.72	major facilitator superfamily protein
Smal_0429	0.20	PAS/PAC sensor-containing diguanylate cyclase/phosphodiesterase
Smal_0430	-23.70	LysR family transcriptional regulator
Smal_0431	-38.20	DoxX family protein
Smal_0435	1.58	hypothetical protein
Smal_0436	6.33	hypothetical protein
Smal_0440	2.34	methionine sulfoxide reductase B
Smal_0441	1.81	hypothetical protein
Smal_0442	0.45	hypothetical protein
Smal_0447	2.11	PAS/PAC sensor hybrid histidine kinase
Smal_0449	35.90	hypothetical protein
Smal_0451	1.70	pseudouridine synthase
Smal_0455	2.71	DNA polymerase IV
Smal_0457	0.30	NAD-dependent epimerase/dehydratase
Smal_0458	0.30	LysR family transcriptional regulator
Smal_0463	0.50	TfoX domain-containing protein

Smal_0464	0.39	putative GAF sensor protein
Smal_0465	0.18	dithiobiotin synthetase
Smal_0466	1.96	GntR family transcriptional regulator
Smal_0468	2.67	cytochrome c biogenesis protein transmembrane region
Smal_0470	0.45	winged helix family two component transcriptional regulator
Smal_0472	0.42	integral membrane sensor signal transduction histidine kinase
Smal_0482	2.98	maleylacetoacetate isomerase
Smal_0483	2.17	twitching motility protein
Smal_0485	2.53	OmpA/MotB domain-containing protein
Smal_0497	1.66	hypothetical protein
Smal_0512	-64.61	GtrA family protein
Smal_0513	0.34	hypothetical protein
Smal_0514	1.81	hypothetical protein
Smal_0515	3.01	hypothetical protein
Smal_0517	1.65	3-oxoacid CoA-transferase subunit A
Smal_0520	2.26	2OG-Fe(II) oxygenase
Smal_0524	1.81	hypothetical protein
Smal_0526	2.14	histone family protein nucleoid-structuring protein H-NS
Smal_0529	0.49	CDP-diacylglycerol/serine O-phosphatidyltransferase
Smal_0534	0.27	hypothetical protein
Smal_0537	2.11	YjgP/YjgQ family permease
Smal_0539	19.23	RDD domain-containing protein
Smal_0540	1.58	site-specific tyrosine recombinase XerD
Smal_0544	0.40	YadA domain-containing protein
Smal_0546	2.03	general secretory pathway protein E
Smal_0549	4.52	general secretion pathway protein H
Smal_0552	-30.24	General secretion pathway protein K
Smal_0558	0.49	family 2 glycosyl transferase
Smal_0567	0.18	bacteriophage N4 adsorption protein B
Smal_0572	1.81	hypothetical protein
Smal_0577	0.45	riboflavin biosynthesis protein RibD
Smal_0579	1.81	hypothetical protein
Smal_0581	2.09	riboflavin synthase subunit alpha
Smal_0582	1.70	3,4-dihydroxy-2-butanone 4-phosphate synthase
Smal_0587	3.01	FAD linked oxidase domain-containing protein
Smal_0588	2.71	membrane-bound metal-dependent hydrolase
Smal_0591	1.81	uroporphyrin-III C/tetrapyrrole (Corrin/Porphyrin) methyltransferase
Smal_0592	1.81	hypothetical protein

Smal_0595	1.66	S-adenosyl-methyltransferase MraW
Smal_0602	2.06	undecaprenyldiphospho-muramoylpentapeptide beta-N- acetylglucosaminyltransferase
Smal_0612	1.81	hypothetical protein
Smal_0614	0.38	5,10-methylenetetrahydrofolate reductase
Smal_0615	-5.64	alpha/beta hydrolase fold domain-containing protein
Smal_0616	9.00	signal transduction histidine kinase LytS
Smal_0617	-14.28	LytTR family two component transcriptional regulator
Smal_0619	2.11	integral membrane sensor signal transduction histidine kinase
Smal_0622	1.81	hypothetical protein
Smal_0624	0.45	hypothetical protein
Smal_0625	-55.18	ybaK/ebsC protein
Smal_0627	1.53	peptidase S9 prolyl oligopeptidase active site domain-containing protein
Smal_0631	0.23	phospholipid/glycerol acyltransferase
Smal_0632	-6.53	metallophosphoesterase
Smal_0633	1.81	membrane-bound metal-dependent hydrolase
Smal_0635	1.81	hypothetical protein
Smal_0641	1.51	Ribokinase
Smal_0645	3.16	TonB-dependent hemoglobin/transferrin/lactoferrin family receptor
Smal_0646	-8.71	hypothetical protein
Smal_0648	2.71	hypothetical protein
Smal_0649	1.51	hypothetical protein
Smal_0650	0.30	ABC transporter-like protein
Smal_0654	0.23	G-D-S-L family lipolytic protein
Smal_0655	0.23	diacylglycerol kinase
Smal_0657	8.14	major facilitator superfamily protein
Smal_0658	38.07	LemA family protein
Smal_0659	0.30	hypothetical protein
Smal_0663	1.64	hypothetical protein
Smal_0664	0.50	Dihydrofolate reductase
Smal_0665	2.26	hypothetical protein
Smal_0666	3.26	diadenosine tetraphosphatase
Smal_0668	1.96	dimethyladenosine transferase
Smal_0674	2.71	hypothetical protein
Smal_0677	3.62	hypothetical protein
Smal_0679	0.27	zinc-binding CMP/dCMP deaminase
Smal_0680	1.51	hypothetical protein
Smal_0681	0.49	hypothetical protein
Smal_0684	1.51	methionine sulfoxide reductase A

Smal_0690	5.51	protein-(glutamine-N5) methyltransferase
Smal_0692	1.51	LysR family transcriptional regulator
Smal_0695	2.26	N-formylglutamate amidohydrolase
Smal_0697	1.51	5'-3' exonuclease
Smal_0698	1.81	nitroreductase
Smal_0703	7.23	RNA polymerase factor sigma-70
Smal_0705	1.99	NAD(P)(+) transhydrogenase
Smal_0709	2.11	hypothetical protein
Smal_0710	3.16	ArsR family transcriptional regulator
Smal_0714	42.27	hypothetical protein
Smal_0715	2.71	flavoprotein WrbA
Smal_0716	0.32	ribonuclease BN/unknown domain fusion protein
Smal_0721	1.51	peptide chain release factor 1
Smal_0724	0.45	outer membrane lipoprotein LolB
Smal_0729	1.87	GTP-dependent nucleic acid-binding protein EngD
Smal_0730	25.94	hypothetical protein
Smal_0732	3.62	hypothetical protein
Smal_0733	1.81	hypothetical protein
Smal_0735	2.03	two component LuxR family transcriptional regulator
Smal_0737	1.81	response regulator receiver modulated diguanylate phosphodiesterase
Smal_0791	2.71	pseudouridine synthase
Smal_0798	-16.29	hypothetical protein
Smal_0799	2.11	zinc-binding alcohol dehydrogenase family protein
Smal_0801	0.27	hypothetical protein
Smal_0806	1.70	L-threonine 3-dehydrogenase
Smal_0811	1.75	colicin V production protein
Smal_0814	1.58	rhs element Vgr protein
Smal_0815	2.11	peptidyl-Asp metalloendopeptidase
Smal_0816	1.71	UDP-2,3-diacylglucosamine hydrolase
Smal_0817	-9.68	PA-phosphatase-like phosphoesterase
Smal_0819	4.84	TonB-dependent receptor plug
Smal_0828	1.52	isocitrate dehydrogenase
Smal_0830	10.40	LysR family transcriptional regulator
Smal_0831	-13.72	hypothetical protein
Smal_0832	0.23	hypothetical protein
Smal_0834	12.83	hypothetical protein
Smal_0839	1.50	histone family protein DNA-binding protein
Smal_0843	1.81	hypothetical protein

Smal_0846	2.03	protein serine/threonine phosphatase
Smal_0848	1.81	hypothetical protein
Smal_0851	1.81	beta-lactamase domain-containing protein
Smal_0852	0.20	hypothetical protein
Smal_0853	1.51	small multidrug resistance protein
Smal_0855	0.45	N-acetyltransferase GCN5
Smal_0859	0.40	recombination protein RecR
Smal_0860	1.96	histidine triad (HIT) protein
Smal_0863	3.16	hypothetical protein
Smal_0864	1.55	ATPase
Smal_0866	16.59	maf protein
Smal_0875	2.49	hypothetical protein
Smal_0876	2.81	aminodeoxychorismate lyase
Smal_0878	1.96	DNA polymerase III subunit delta'
Smal_0880	1.81	4-oxalocrotonate tautomerase
Smal_0881	2.71	late control D family protein
Smal_0882	1.81	tail X family protein
Smal_0883	2.71	P2 GpU family protein
Smal_0886	0.30	phage major tail tube protein
Smal_0888	-15.84	hypothetical protein
Smal_0889	1.73	hypothetical protein
Smal_0890	-16.05	phage tail protein I
Smal_0891	0.15	baseplate J family protein
Smal_0892	6.33	GPW/gp25 family protein
Smal_0893	1.81	phage baseplate assembly protein V
Smal_0894	0.23	hypothetical protein
Smal_0896	9.73	lysozyme
Smal_0897	13.49	hypothetical protein
Smal_0898	10.51	hypothetical protein
Smal_0899	0.36	hypothetical protein
Smal_0900	1.81	hypothetical protein
Smal_0901	-23.00	hypothetical protein
Smal_0902	-18.98	hypothetical protein
Smal_0903	0.45	virulence-associated E family protein
Smal_0906	35.85	hypothetical protein
Smal_0907	0.30	TonB-dependent siderophore receptor
Smal_0908	1.81	PepSY-associated TM helix domain-containing protein
Smal_0911	2.26	pentapeptide repeat-containing protein

Smal_0914	2.71	hypothetical protein
Smal_0915	0.45	LysR family transcriptional regulator
Smal_0916	1.81	major facilitator superfamily protein
Smal_0917	0.30	TonB-dependent siderophore receptor
Smal_0918	0.45	integral membrane sensor signal transduction histidine kinase
Smal_0919	1.81	winged helix family two component transcriptional regulator
Smal_0923	0.45	hypothetical protein
Smal_0924	0.14	hypothetical protein
Smal_0925	17.65	major facilitator superfamily protein
Smal_0926	2.71	MerR family transcriptional regulator
Smal_0927	1.81	short chain dehydrogenase
Smal_0928	-17.90	LysR family transcriptional regulator
Smal_0929	2.11	pyrroline-5-carboxylate reductase
Smal_0933	4.52	aldo/keto reductase
Smal_0936	0.30	N-acetyltransferase GCN5
Smal_0941	1.94	Holliday junction resolvase-like protein
Smal_0942	1.71	aspartate carbamoyltransferase catalytic subunit
Smal_0943	9.61	hypothetical protein
Smal_0945	0.36	hypothetical protein
Smal_0959	2.86	KpsF/GutQ family protein
Smal_0960	1.73	BolA family protein
Smal_0965	0.13	phosphoribosylglycinamide formyltransferase
Smal_0967	-24.74	hypothetical protein
Smal_0983	3.32	flavodoxin/nitric oxide synthase
Smal_0984	9.72	ApbE family lipoprotein
Smal_0985	4.22	hypothetical protein
Smal_0986	-20.15	hypothetical protein
Smal_0987	0.45	PepSY-associated TM helix domain-containing protein
Smal_0989	23.03	hypothetical protein
Smal_0991	1.51	Sel1 domain-containing protein repeat-containing protein
Smal_0993	-38.76	hypothetical protein
Smal_1005	0.30	hypothetical protein
Smal_1006	1.81	hypothetical protein
Smal_1008	2.26	OsmC family protein
Smal_1009	2.26	phenazine biosynthesis protein PhzF family
Smal_1010	0.23	phenazine biosynthesis protein PhzF family
Smal_1014	2.71	hypothetical protein
Smal_1017	-6.47	alpha amylase catalytic protein

Smal_1019	4.60	alpha-glucosidase
Smal_1020	0.45	hypothetical protein
Smal_1021	0.30	major facilitator superfamily protein
Smal_1024	0.30	N-acetyltransferase GCN5
Smal_1027	16.45	hypothetical protein
Smal_1032	0.45	LacI family transcriptional regulator
Smal_1038	0.45	hypothetical protein
Smal_1039	-7.17	short-chain dehydrogenase/reductase SDR
Smal_1041	1.81	Xylose isomerase domain-containing protein
Smal_1044	-42.15	hypothetical protein
Smal_1045	-17.64	cytochrome c class I
Smal_1046	1.81	Xylose isomerase domain-containing protein
Smal_1047	4.07	oxidoreductase domain-containing protein
Smal_1048	0.34	nucleoside:H symporter
Smal_1050	1.81	LacI family transcriptional regulator
Smal_1054	0.30	isocitrate lyase family protein
Smal_1056	2.11	diguanylate cyclase
Smal_1058	2.71	hypothetical protein
Smal_1061	2.03	hypothetical protein
Smal_1063	-8.98	hypothetical protein
Smal_1067	2.26	metallophosphoesterase
Smal_1068	-12.73	hypothetical protein
Smal_1069	2.71	hypothetical protein
Smal_1072	-14.66	hypothetical protein
Smal_1073	1.63	GntR family transcriptional regulator
Smal_1077	-8.14	phosphoribosyl-dephospho-CoA transferase
Smal_1078	0.45	malonate decarboxylase subunit gamma
Smal_1079	3.01	malonate decarboxylase subunit beta
Smal_1082	-2.38	TonB-dependent siderophore receptor
Smal_1083	0.50	malate:quinone oxidoreductase
Smal_1084	-70.23	N-acetyltransferase GCN5
Smal_1089	-18.19	hypothetical protein
Smal_1090	1.81	OPT family oligopeptide transporter
Smal_1092	2.71	hypothetical protein
Smal_1096	1.65	septum formation inhibitor
Smal_1098	2.71	integral membrane sensor signal transduction histidine kinase
Smal_1099	1.66	two component LuxR family transcriptional regulator
Smal_1102	0.23	peptidase M15B and M15C DD-carboxypeptidase VanY/endolysin

Smal_1104	-29.16	hypothetical protein
Smal_1105	-29.16	hypothetical protein
Smal_1106	0.45	hypothetical protein
Smal_1107	1.96	phosphoribosylglycinamide formyltransferase 2
Smal_1109	2.03	arginyl-tRNA-protein transferase
Smal_1110	8.38	hypothetical protein
Smal_1111	0.36	ECF subfamily RNA polymerase sigma-24 subunit
Smal_1114	8.14	enoyl-CoA hydratase/isomerase
Smal_1119	1.64	50S ribosomal protein L21
Smal_1120	1.52	50S ribosomal protein L27
Smal_1129	1.81	hypothetical protein
Smal_1130	2.26	hypothetical protein
Smal_1131	0.30	hypothetical protein
Smal_1132	0.39	TonB-dependent receptor plug
Smal_1136	2.71	NAD-binding D-isomer specific 2-hydroxyacid dehydrogenase
Smal_1139	3.62	hypothetical protein
Smal_1144	3.16	diguanylate cyclase/phosphodiesterase
Smal_1148	0.45	hypothetical protein
Smal_1149	1.51	cytochrome c assembly protein
Smal_1151	1.81	2-nitropropane dioxygenase
Smal_1154	1.95	16S rRNA-processing protein RimM
Smal_1155	1.63	tRNA (guanine-N(1)-)-methyltransferase
Smal_1158	0.23	hypothetical protein
Smal_1160	0.23	MATE efflux family protein
Smal_1164	2.49	hypothetical protein
Smal_1168	36.13	putative barnase inhibitor
Smal_1176	7.61	lysine exporter protein LysE/YggA
Smal_1177	2.71	hypothetical protein
Smal_1184	2.51	2,3-diketo-5-methylthio-1-phosphopentane phosphatase
Smal_1185	0.36	DegT/DnrJ/EryC1/StrS aminotransferase
Smal_1186	0.23	hypothetical protein
Smal_1188	0.50	alpha/beta hydrolase fold domain-containing protein
Smal_1189	1.51	integral membrane sensor signal transduction histidine kinase
Smal_1191	5.43	metallophosphoesterase
Smal_1192	0.45	HxlR family transcriptional regulator
Smal_1193	1.81	major facilitator superfamily protein
Smal_1198	1.50	phosphoglyceromutase
Smal_1200	0.18	metal dependent phosphohydrolase

Smal_1203	4.52	hypothetical protein
Smal_1207	2.71	hypothetical protein
Smal_1208	1.68	peptidase M28
Smal_1212	0.45	major facilitator superfamily protein
Smal_1214	0.49	TonB-dependent receptor
Smal_1217	1.58	Nucleotide diphosphatase
Smal_1218	-21.26	methylated-DNA--protein-cysteine methyltransferase
Smal_1219	1.81	AraC family transcriptional regulator
Smal_1222	3.62	hypothetical protein
Smal_1224	0.27	hypothetical protein
Smal_1226	2.11	PAS/PAC sensor-containing diguanylate cyclase/phosphodiesterase
Smal_1227	1.51	diguanylate cyclase
Smal_1229	0.10	diguanylate cyclase
Smal_1230	1.66	SMC domain-containing protein
Smal_1231	1.51	hypothetical protein
Smal_1232	5.81	hypothetical protein
Smal_1233	-11.78	Glyoxalase/bleomycin resistance protein/dioxygenase
Smal_1234	0.39	beta-lactamase
Smal_1235	1.60	ABC transporter-like protein
Smal_1236	0.45	glucose-1-phosphatase/inositol phosphatase
Smal_1238	39.78	glutathione-dependent formaldehyde-activating protein
Smal_1242	0.45	hypothetical protein
Smal_1248	2.26	hypothetical protein
Smal_1249	1.68	acetyl-CoA carboxylase carboxyltransferase subunit alpha
Smal_1252	0.36	Ribonuclease H
Smal_1264	1.67	diguanylate cyclase
Smal_1267	0.45	pili assembly chaperone
Smal_1268	1.81	hypothetical protein
Smal_1271	-14.22	pili assembly chaperone
Smal_1272	0.15	Spore coat U domain-containing protein
Smal_1279	0.15	Sel1 domain-containing protein repeat-containing protein
Smal_1282	1.81	peptidase S45 penicillin amidase
Smal_1286	0.30	MarR family transcriptional regulator
Smal_1288	2.26	secretion protein HlyD family protein
Smal_1289	1.81	EmrB/QacA subfamily drug resistance transporter
Smal_1294	0.45	glutathione-dependent formaldehyde-activating protein
Smal_1296	0.30	NodT family RND efflux system outer membrane lipoprotein
Smal_1298	0.30	RND family efflux transporter MFP subunit

Smal_1302	1.53	ribonuclease T
Smal_1304	-13.83	hypothetical protein
Smal_1307	-36.92	phosphate uptake regulator PhoU
Smal_1308	0.45	phosphate transporter ATP-binding protein
Smal_1309	-12.10	phosphate ABC transporter permease
Smal_1310	0.18	phosphate ABC transporter permease
Smal_1311	1.81	phosphate ABC transporter substrate-binding protein
Smal_1312	4.65	phosphate ABC transporter substrate-binding protein
Smal_1313	2.71	phosphate-selective porin O and P
Smal_1315	1.51	hypothetical protein
Smal_1316	0.45	enoyl-CoA hydratase/isomerase
Smal_1320	4.52	N-acetylmuramyl-L-alanine amidase, negative regulator of AmpC, AmpD
Smal_1321	0.45	hypothetical protein
Smal_1323	2.71	hypothetical protein
Smal_1325	15.03	hypothetical protein
Smal_1327	7.34	hypothetical protein
Smal_1331	7.36	hypothetical protein
Smal_1336	2.41	hypothetical protein
Smal_1338	0.45	FAD dependent oxidoreductase
Smal_1339	0.45	peptidase C26
Smal_1341	1.51	family 1 extracellular solute-binding protein
Smal_1343	0.27	spermidine/putrescine ABC transporter ATPase
Smal_1345	0.30	ornithine carbamoyltransferase
Smal_1350	-28.12	hypothetical protein
Smal_1351	0.30	hypothetical protein
Smal_1355	1.51	ABC transporter-like protein
Smal_1357	3.11	(dimethylallyl)adenosine tRNA methylthiotransferase
Smal_1358	7.61	glutathione S-transferase domain-containing protein
Smal_1360	0.45	Lytic transglycosylase
Smal_1364	1.55	glutathione S-transferase domain-containing protein
Smal_1367	4.72	hypothetical protein
Smal_1369	1.81	hypothetical protein
Smal_1372	6.33	alkylhydroperoxidase-like protein
Smal_1375	0.33	short-chain dehydrogenase/reductase SDR
Smal_1378	1.51	putative type IV pilus assembly protein FimT
Smal_1380	2.11	hypothetical protein
Smal_1381	3.16	PilX protein
Smal_1390	33.72	hypothetical protein

Smal_1395	1.81	DNA internalization-related competence protein ComEC/Rec2
Smal_1397	4.82	biopolymer transport protein ExbD/TolR
Smal_1398	2.11	lipid A ABC exporter, fused ATPase and inner membrane subunits MsbA
Smal_1399	5.43	tetraacyldisaccharide 4'-kinase
Smal_1400	1.91	3-deoxy-manno-octulosonate cytidylyltransferase
Smal_1401	2.07	protein tyrosine phosphatase
Smal_1402	1.58	NHL repeat containing protein
Smal_1406	1.81	hypothetical protein
Smal_1409	1.81	ABC transporter-like protein
Smal_1414	0.30	hypothetical protein
Smal_1415	0.45	ArsR family transcriptional regulator
Smal_1416	0.45	hypothetical protein
Smal_1417	1.81	hypothetical protein
Smal_1418	47.20	hypothetical protein
Smal_1419	0.45	hypothetical protein
Smal_1423	11.77	hypothetical protein
Smal_1430	2.03	BLUF domain-containing protein
Smal_1433	5.43	N-acetyltransferase GCN5
Smal_1435	1.58	FKBP-type peptidylprolyl isomerase
Smal_1436	2.71	hypothetical protein
Smal_1438	1.60	luciferase-like monooxygenase
Smal_1442	1.76	hypothetical protein
Smal_1444	0.15	peptidase S10 serine carboxypeptidase
Smal_1451	1.91	hypothetical protein
Smal_1453	1.99	cell division protein FtsB
Smal_1462	-5.41	transposase IS116/IS110/IS902 family protein
Smal_1463	2.71	hypothetical protein
Smal_1468	1.81	hypothetical protein
Smal_1470	2.11	tRNA delta(2)-isopentenylpyrophosphate transferase
Smal_1474	0.32	2-polyprenylphenol 6-hydroxylase
Smal_1479	0.36	carbon starvation protein CstA
Smal_1480	0.49	TonB-dependent receptor
Smal_1482	2.17	TonB-dependent siderophore receptor
Smal_1484	0.30	TonB-dependent receptor plug
Smal_1486	-7.69	hypothetical protein
Smal_1487	2.71	hypothetical protein
Smal_1488	0.15	2-oxoacid dehydrogenase subunit E1
Smal_1493	2.71	alkaline phosphatase

Smal_1501	0.45	TonB-dependent siderophore receptor
Smal_1502	0.18	Pirin domain-containing protein
Smal_1503	2.26	LysR family transcriptional regulator
Smal_1509	1.54	alkyl hydroperoxide reductase
Smal_1510	1.53	amino acid-binding ACT domain-containing protein
Smal_1512	1.96	hypothetical protein
Smal_1514	0.39	sugar transporter
Smal_1519	2.26	hypothetical protein
Smal_1522	1.51	two component Fis family transcriptional regulator
Smal_1526	1.70	two component LuxR family transcriptional regulator
Smal_1527	0.40	keto-hydroxyglutarate-aldolase/keto-deoxy- phosphogluconate aldolase
Smal_1531	1.54	glucose-6-phosphate 1-dehydrogenase
Smal_1533	0.23	folate-binding protein YgfZ
Smal_1540	0.45	hypothetical protein
Smal_1549	0.45	methyltransferase
Smal_1554	2.26	beta-lactamase domain-containing protein
Smal_1556	1.71	uracil phosphoribosyltransferase
Smal_1558	1.81	NHL repeat containing protein
Smal_1559	-15.27	metallophosphoesterase
Smal_1562	4.22	deoxyribodipyrimidine photo-lyase
Smal_1565	4.52	hypothetical protein
Smal_1566	1.81	short-chain dehydrogenase/reductase SDR
Smal_1568	2.71	hydrophobe/amphiphile efflux-1 (HAE1) family transporter
Smal_1569	-7.08	short-chain dehydrogenase/reductase SDR
Smal_1570	0.13	NodT family RND efflux system outer membrane lipoprotein
Smal_1572	1.81	endonuclease/exonuclease/phosphatase
Smal_1573	4.52	cytochrome P450
Smal_1574	-22.12	hypothetical protein
Smal_1575	1.81	LuxR family transcriptional regulator
Smal_1576	0.23	chaperone protein HchA
Smal_1583	-8.39	secretion protein HlyD family protein
Smal_1584	9.95	ABC transporter-like protein
Smal_1585	2.71	abortive infection protein
Smal_1587	1.81	hypothetical protein
Smal_1588	5.43	N-acetyltransferase GCN5
Smal_1590	1.86	SsrA-binding protein
Smal_1591	1.81	cyclase/dehydrase
Smal_1593	1.70	SmpA/OmlA domain-containing protein

Smal_1595	-8.31	G-D-S-L family lipolytic protein
Smal_1605	1.81	prephenate dehydrogenase
Smal_1606	0.30	hypothetical protein
Smal_1609	0.18	hypothetical protein
Smal_1612	2.49	hypothetical protein
Smal_1614	1.81	S-adenosylmethionine--tRNA ribosyltransferase-isomerase
Smal_1618	1.51	preprotein translocase subunit SecF
Smal_1622	6.33	YaeQ family protein
Smal_1623	4.52	pseudouridine synthase
Smal_1624	1.81	hypothetical protein
Smal_1630	-4.84	hypothetical protein
Smal_1631	-11.93	LysR family transcriptional regulator
Smal_1633	4.89	transposase IS116/IS110/IS902 family protein
Smal_1634	0.45	cytochrome c class I
Smal_1637	2.41	acriflavin resistance protein
Smal_1638	0.39	acriflavin resistance protein
Smal_1640	1.67	N-acetyltransferase GCN5
Smal_1642	1.70	Agmatine deiminase
Smal_1643	1.96	hypothetical protein
Smal_1648	1.81	hypothetical protein
Smal_1649	1.64	hypothetical protein
Smal_1660	1.54	hypothetical protein
Smal_1663	-6.68	hypothetical protein
Smal_1664	7.50	lysine exporter protein LysE/YggA
Smal_1668	1.51	potassium efflux system protein
Smal_1674	0.44	hypothetical protein
Smal_1677	26.52	hypothetical protein
Smal_1679	-12.44	ThiJ/PfpI domain-containing protein
Smal_1680	-14.17	hypothetical protein
Smal_1685	1.81	Activator of Hsp90 ATPase 1 family protein
Smal_1687	-12.44	short chain dehydrogenase
Smal_1688	1.81	AraC family transcriptional regulator
Smal_1692	0.45	AraC family transcriptional regulator
Smal_1693	3.62	TonB-dependent receptor
Smal_1694	-17.97	FMN reductase
Smal_1697	7.03	flavin-dependent oxidoreductase
Smal_1698	-4.28	amidohydrolase
Smal_1699	-9.47	N-acetyltransferase GCN5

Smal_1700	5.41	polar amino acid ABC transporter inner membrane subunit
Smal_1705	2.17	short-chain dehydrogenase/reductase SDR
Smal_1707	0.45	short-chain dehydrogenase/reductase SDR
Smal_1708	1.81	hypothetical protein
Smal_1709	2.26	TetR family transcriptional regulator
Smal_1712	2.26	major facilitator superfamily protein
Smal_1713	1.58	short-chain dehydrogenase/reductase SDR
Smal_1714	5.43	putative acetyltransferase
Smal_1715	1.51	ArsR family transcriptional regulator
Smal_1718	0.45	DoxX family protein
Smal_1722	0.36	hypothetical protein
Smal_1726	0.27	hypothetical protein
Smal_1729	1.96	dihydrodipicolinate synthetase
Smal_1735	2.26	response regulator receiver protein
Smal_1736	4.52	PAS/PAC sensor signal transduction histidine kinase
Smal_1738	2.26	CheR-type MCP methyltransferase
Smal_1740	0.38	response regulator receiver sensor signal transduction histidine kinase
Smal_1743	2.11	hypothetical protein
Smal_1747	1.81	AraC family transcriptional regulator
Smal_1748	2.71	hypothetical protein
Smal_1749	0.34	bifunctional aspartokinase I/homoserine dehydrogenase I
Smal_1751	0.30	threonine synthase
Smal_1757	1.58	histidinol dehydrogenase
Smal_1760	0.23	imidazole glycerol phosphate synthase subunit HisH
Smal_1762	2.35	imidazole glycerol phosphate synthase subunit HisF
Smal_1764	2.03	metallophosphoesterase
Smal_1765	0.45	hypothetical protein
Smal_1766	2.49	glucokinase
Smal_1772	2.71	heavy metal transport/detoxification protein
Smal_1775	1.85	LacI family transcriptional regulator
Smal_1781	1.58	Acireductone dioxygenase ARD
Smal_1783	0.30	hypothetical protein
Smal_1785	2.20	amino acid permease-associated protein
Smal_1790	2.03	hypothetical protein
Smal_1792	2.11	winged helix family two component transcriptional regulator
Smal_1795	-39.95	hypothetical protein
Smal_1801	0.45	Glyoxalase/bleomycin resistance protein/dioxygenase
Smal_1802	0.39	enoyl-CoA hydratase/isomerase

Smal_1803	1.81	FeoA family protein
Smal_1807	8.12	NAD(P)H dehydrogenase (quinone)
Smal_1808	1.81	MerR family transcriptional regulator
Smal_1810	4.52	dihydrodipicolinate reductase
Smal_1815	0.23	hypothetical protein
Smal_1819	2.41	Vault protein inter-alpha-trypsin domain-containing protein
Smal_1821	2.71	hypothetical protein
Smal_1822	26.17	response regulator receiver protein
**Smal_1830**	**16.30**	**enoyl-CoA hydratase (rpfF)**
Smal_1833	1.71	betaine aldehyde dehydrogenase
Smal_1835	0.34	choline/carnitine/betaine transporter
Smal_1837	42.15	AbrB family transcriptional regulator
Smal_1838	2.58	hypothetical protein
Smal_1840	0.42	TonB family protein
Smal_1841	0.45	TonB family protein
Smal_1842	17.64	alpha/beta hydrolase fold domain-containing protein
Smal_1843	2.71	chemotaxis-specific methylesterase
Smal_1845	2.71	CheR-type MCP methyltransferase
Smal_1846	6.33	methyl-accepting chemotaxis sensory transducer
Smal_1847	1.71	methyl-accepting chemotaxis sensory transducer
Smal_1849	4.52	CheW protein
Smal_1850	1.81	YcgR family protein
Smal_1851	1.81	methyl-accepting chemotaxis sensory transducer
Smal_1853	1.81	response regulator receiver protein
Smal_1855	2.71	CheW protein
Smal_1856	2.71	Cobyrinic acid ac-diamide synthase
Smal_1857	4.07	flagellar motor protein MotD
Smal_1858	9.95	flagellar motor protein
Smal_1859	1.81	CheA signal transduction histidine kinase
Smal_1861	1.81	response regulator receiver protein
Smal_1863	3.16	Cobyrinic acid ac-diamide synthase
Smal_1864	1.66	flagellar biosynthesis regulator FlhF
Smal_1865	2.03	flagellar biosynthesis protein FlhA
Smal_1867	8.86	diguanylate cyclase/phosphodiesterase
Smal_1868	17.90	flagellar biosynthetic protein FliR
Smal_1869	35.10	export protein FliQ family 3
Smal_1870	1.58	flagellar biosynthesis protein FliP
Smal_1872	28.18	flagellar motor switch protein FliN

Smal_1873	2.41	flagellar motor switch protein FliM
Smal_1874	4.52	flagellar basal body-associated protein FliL
Smal_1875	0.45	flagellar hook-length control protein
Smal_1876	2.26	flagellar export protein FliJ
Smal_1877	17.04	FliI/YscN family ATPase
Smal_1878	1.81	flagellar assembly protein FliH
Smal_1879	2.26	flagellar motor switch protein FliG
Smal_1881	12.93	flagellar hook-basal body complex subunit FliE
Smal_1883	1.56	Fis family sigma-54 specific transcriptional regulator
Smal_1884	1.81	response regulator receiver protein
Smal_1885	10.85	RNA polymerase sigma-54 subunit RpoN
Smal_1890	1.81	hypothetical protein
Smal_1891	-50.19	flagellar protein FliS
Smal_1892	2.26	flagellar hook-associated 2 domain-containing protein
Smal_1893	2.95	flagellin
Smal_1894	3.13	flagellin
Smal_1895	2.30	flagellin
Smal_1896	3.01	flagellar hook-associated protein FlgL
Smal_1897	3.05	flagellar hook-associated protein FlgK
Smal_1898	2.11	flagellar rod assembly protein/muramidase FlgJ
Smal_1903	1.87	flagellar hook protein FlgE
Smal_1906	1.81	flagellar basal body rod protein FlgB
Smal_1907	15.00	response regulator receiver modulated CheW protein
Smal_1908	4.52	flagellar basal body P-ring biosynthesis protein FlgA
Smal_1909	2.41	putative anti-sigma-28 factor FlgM
Smal_1914	6.33	PAS/PAC sensor-containing diguanylate cyclase/phosphodiesterase
Smal_1915	35.83	hypothetical protein
Smal_1916	15.03	hypothetical protein
Smal_1918	1.81	hypothetical protein
Smal_1919	1.81	tRNA-specific 2-thiouridylase MnmA
Smal_1920	1.99	NUDIX hydrolase
Smal_1922	1.51	ATP-dependent Clp protease adaptor protein ClpS
Smal_1926	-38.38	hypothetical protein
Smal_1928	2.26	leucyl/phenylalanyl-tRNA--protein transferase
Smal_1929	3.62	hypothetical protein
Smal_1931	1.58	cell wall hydrolase/autolysin
Smal_1932	1.58	thioredoxin reductase
Smal_1937	4.52	hypothetical protein

Smal_1938	25.24	hypothetical protein
Smal_1939	1.81	putative esterase
Smal_1940	1.51	hypothetical protein
Smal_1942	-5.00	transport system permease
Smal_1946	1.81	hypothetical protein
Smal_1951	4.40	type 11 methyltransferase
Smal_1952	-6.65	hypothetical protein
Smal_1954	3.75	cyclopropane-fatty-acyl-phospholipid synthase
Smal_1956	0.45	amine oxidase
Smal_1957	-5.49	stearoyl-CoA 9-desaturase
Smal_1959	7.58	RNA polymerase sigma factor RpoE
Smal_1961	-38.21	hypothetical protein
Smal_1965	-8.59	NAD(P)H dehydrogenase (quinone)
Smal_1967	0.45	LysR family transcriptional regulator
Smal_1968	1.81	Pirin domain-containing protein
Smal_1971	1.71	putative cointegrate resolution protein T
Smal_1973	0.50	hypothetical protein
Smal_1979	1.81	ArsR family transcriptional regulator
Smal_1981	2.08	arsenical-resistance protein
Smal_1984	-29.38	protein tyrosine phosphatase
Smal_1994	-10.14	OsmC family protein
Smal_1995	6.15	NmrA family protein
Smal_1996	1.81	LysR family transcriptional regulator
Smal_1997	-16.95	ECF subfamily RNA polymerase sigma-24 subunit
Smal_2000	0.45	ECF subfamily RNA polymerase sigma-24 subunit
Smal_2001	0.45	beta-lactamase domain-containing protein
Smal_2002	0.36	hypothetical protein
Smal_2003	0.39	TetR family transcriptional regulator
Smal_2005	1.68	TetR family transcriptional regulator
Smal_2008	1.53	alcohol dehydrogenase zinc-binding domain-containing protein
Smal_2011	0.45	major facilitator superfamily protein
Smal_2012	1.81	aldo/keto reductase
Smal_2013	1.81	cupin
Smal_2015	0.23	PepSY-associated TM helix domain-containing protein
Smal_2016	0.30	TonB-dependent siderophore receptor
Smal_2017	1.81	anti-FecI sigma factor FecR
Smal_2019	13.88	hypothetical protein
Smal_2020	46.05	hypothetical protein

Smal_2023	0.45	acriflavin resistance protein
Smal_2024	3.08	outer membrane efflux protein
Smal_2026	7.20	TetR family transcriptional regulator
Smal_2028	1.81	hypothetical protein
Smal_2029	1.81	LacI family transcriptional regulator
Smal_2032	2.51	PTS system fructose subfamily transporter subunit IIC
Smal_2033	1.81	carbohydrate-selective porin OprB
Smal_2034	1.81	beta-Ig-H3/fasciclin
Smal_2035	0.45	hypothetical protein
Smal_2036	5.65	peptidase S1 and S6 chymotrypsin/Hap
Smal_2037	-56.33	hypothetical protein
Smal_2040	-17.97	FMN reductase
Smal_2041	0.45	hypothetical protein
Smal_2042	0.45	5- methyltetrahydropteroyltriglutamate/homocysteine S-methyltransferase
Smal_2043	0.30	hypothetical protein
Smal_2045	1.81	Arsenical pump membrane protein
Smal_2047	5.87	TonB-dependent receptor
Smal_2049	3.48	multi- copper enzyme maturation ABC transporter permease-like protein
Smal_2052	60.60	hypothetical protein
Smal_2053	29.32	hypothetical protein
Smal_2056	9.40	PHB depolymerase family esterase
Smal_2058	10.75	alpha/beta hydrolase fold domain-containing protein
Smal_2059	9.73	MaoC domain-containing protein dehydratase
Smal_2063	-10.63	hypothetical protein
Smal_2064	1.70	GntR family transcriptional regulator
Smal_2065	0.45	TonB-dependent receptor
Smal_2066	2.26	alginate lyase
Smal_2068	-8.01	major facilitator superfamily protein
Smal_2070	2.71	G-D-S-L family lipolytic protein
Smal_2071	3.62	PfkB domain-containing protein
Smal_2074	1.81	peptidase S15
Smal_2076	6.38	DNA/RNA non-specific endonuclease
Smal_2077	3.62	HAD-superfamily hydrolase
Smal_2078	41.05	hypothetical protein
Smal_2080	-2.11	CheR-type MCP methyltransferase
Smal_2082	2.71	LysR family transcriptional regulator
Smal_2083	9.08	hypothetical protein
Smal_2085	35.23	hypothetical protein

Smal_2086	15.33	hypothetical protein
Smal_2087	-3.85	Fis family sigma-54 specific transcriptional regulator
Smal_2088	8.86	hypothetical protein
Smal_2089	22.95	hypothetical protein
Smal_2092	-6.97	hypothetical protein
Smal_2093	10.96	hypothetical protein
Smal_2095	4.41	NUDIX hydrolase
Smal_2096	-36.00	RebB protein
Smal_2097	-19.41	RebB protein
Smal_2099	-19.19	RebB protein
Smal_2100	-22.12	hypothetical protein
Smal_2104	40.00	hypothetical protein
Smal_2105	0.45	putative sigma-54 specific transcriptional regulator
Smal_2109	-23.30	protein tyrosine/serine phosphatase
Smal_2110	-2.27	TonB-dependent receptor
Smal_2111	-8.11	TetR family transcriptional regulator
Smal_2112	-8.94	hypothetical protein
Smal_2114	0.26	PAS/PAC sensor-containing diguanylate cyclase
Smal_2115	0.41	ABC transporter-like protein
Smal_2116	0.23	RND family efflux transporter MFP subunit
Smal_2121	2.71	hypothetical protein
Smal_2122	-12.49	Siderophore-interacting protein
Smal_2123	1.51	TonB-dependent siderophore receptor
Smal_2124	-17.46	hypothetical protein
Smal_2125	0.36	PepSY-associated TM helix domain-containing protein
Smal_2126	-18.78	hypothetical protein
Smal_2127	0.39	hypothetical protein
Smal_2128	1.81	putative TetR family transcriptional regulator
Smal_2129	0.49	TonB-dependent siderophore receptor
Smal_2132	10.85	conjugal transfer protein TrbP
Smal_2134	-17.46	hypothetical protein
Smal_2135	2.26	hypothetical protein
Smal_2138	1.63	integral membrane sensor signal transduction histidine kinase
Smal_2139	2.41	two component LuxR family transcriptional regulator
Smal_2140	4.07	hypothetical protein
Smal_2141	4.03	low temperature requirement A
Smal_2142	-19.62	hypothetical protein
Smal_2145	0.36	TonB-dependent receptor

Smal_2146	2.26	beta-lactamase
Smal_2147	0.45	hypothetical protein
Smal_2150	8.81	hypothetical protein
Smal_2152	1.99	TetR family transcriptional regulator
Smal_2154	1.81	hypothetical protein
Smal_2155	-16.84	hypothetical protein
Smal_2158	-7.68	ABC transporter-like protein
Smal_2161	2.16	TonB-dependent receptor
Smal_2166	3.45	heavy metal sensor signal transduction histidine kinase
Smal_2169	3.07	CzcA family heavy metal efflux pump
Smal_2175	1.81	Thioredoxin domain
Smal_2176	2.20	hypothetical protein
Smal_2177	-33.04	hypothetical protein
Smal_2181	2.26	hypothetical protein
Smal_2183	0.30	response regulator receiver protein
Smal_2184	3.25	hypothetical protein
Smal_2185	0.45	hypothetical protein
Smal_2188	37.54	ECF subfamily RNA polymerase sigma-24 subunit
Smal_2193	-13.42	OsmC family protein
Smal_2194	-7.49	ABC transporter-like protein
Smal_2196	5.41	hypothetical protein
Smal_2197	6.73	hypothetical protein
Smal_2198	11.43	biopolymer transport protein ExbD/TolR
Smal_2203	10.58	general secretion pathway protein H
Smal_2205	-17.00	type II secretion system protein J
Smal_2207	3.62	alkaline phosphatase
Smal_2208	0.30	alkaline phosphatase
Smal_2209	0.45	general secretion pathway protein F
Smal_2210	0.45	general secretory pathway protein E
Smal_2211	-8.77	general secretion pathway protein D
Smal_2213	0.45	general secretion pathway protein L
Smal_2214	10.43	General secretion pathway protein K
Smal_2215	54.01	general secretion pathway protein G
Smal_2216	3.62	hypothetical protein
Smal_2217	-9.47	hypothetical protein
Smal_2218	0.45	Fe2+-dicitrate sensor, membrane protein
Smal_2220	6.94	Secretin/TonB short domain
Smal_2221	1.81	AsnC family transcriptional regulator

Smal_2226	0.23	ABC transporter-like protein
Smal_2227	2.03	molybdate ABC transporter inner membrane subunit
Smal_2229	1.51	Crp/Fnr family transcriptional regulator
Smal_2234	1.79	nitrate reductase molybdenum cofactor assembly chaperone
Smal_2241	1.65	sulfur transfer protein ThiS
Smal_2245	1.81	ribonucleotide reductase subunit alpha
Smal_2247	-27.92	hypothetical protein
Smal_2251	0.34	YD repeat-containing protein
Smal_2252	-6.57	AraC family transcriptional regulator
Smal_2253	0.15	fusaric acid resistance protein
Smal_2255	5.34	secretion protein HlyD family protein
Smal_2257	1.68	hypothetical protein
Smal_2258	1.81	hypothetical protein
Smal_2259	0.30	hypothetical protein
Smal_2260	7.23	hypothetical protein
Smal_2261	0.30	hypothetical protein
Smal_2262	1.81	diacylglycerol kinase
Smal_2263	1.81	hypothetical protein
Smal_2264	4.52	hypothetical protein
Smal_2266	0.45	hypothetical protein
Smal_2267	-13.85	hypothetical protein
Smal_2269	-4.50	TonB-dependent receptor
Smal_2270	19.40	hypothetical protein
Smal_2271	0.45	short-chain dehydrogenase/reductase SDR
Smal_2272	2.71	enterobactin synthase subunit F
Smal_2273	18.37	phosphopantetheine-binding protein
Smal_2275	-9.48	AMP-dependent synthetase and ligase
Smal_2278	1.81	inner membrane protein
Smal_2280	2.71	N-acetyltransferase GCN5
Smal_2281	0.18	Thioredoxin domain
Smal_2289	-12.09	TonB-dependent receptor plug
Smal_2290	0.30	alkyl hydroperoxide reductase
Smal_2293	5.43	Thioredoxin domain
Smal_2299	1.64	choline/carnitine/betaine transporter
Smal_2301	-19.92	ECF subfamily RNA polymerase sigma-24 subunit
Smal_2302	-11.06	anti-FecI sigma factor FecR
Smal_2303	0.30	TonB-dependent receptor
Smal_2304	43.84	small multidrug resistance protein

Smal_2305	5.43	small multidrug resistance protein
Smal_2308	1.81	TonB-dependent siderophore receptor
Smal_2312	1.58	type 11 methyltransferase
Smal_2313	-86.43	hypothetical protein
Smal_2315	-2.03	YadA domain-containing protein
Smal_2317	-54.66	Flp/Fap pilin component
Smal_2318	-9.58	peptidase A24A prepilin type IV
Smal_2320	4.55	Flp pilus assembly protein CpaB
Smal_2321	0.45	type II and III secretion system protein
Smal_2324	-5.46	type II secretion system protein
Smal_2325	5.05	type II secretion system protein
Smal_2327	-23.73	hypothetical protein
Smal_2330	24.65	hypothetical protein
Smal_2331	5.73	alpha/beta hydrolase fold domain-containing protein
Smal_2332	2.86	hypothetical protein
Smal_2333	3.68	hypothetical protein
Smal_2334	-5.30	amidohydrolase
Smal_2335	-12.28	DoxX family protein
Smal_2340	-23.98	two component LuxR family transcriptional regulator
Smal_2341	-7.93	BAAT/Acyl-CoA thioester hydrolase
Smal_2343	0.45	histidine kinase internal region
Smal_2344	3.62	amidohydrolase
Smal_2345	6.73	ankyrin
Smal_2346	3.62	LysR family transcriptional regulator
Smal_2347	-35.84	hypothetical protein
Smal_2348	0.30	cytosine deaminase
Smal_2351	64.96	hypothetical protein
Smal_2352	3.62	hypothetical protein
Smal_2353	-5.74	TonB-dependent receptor
Smal_2355	2.71	CDP-diacylglycerol pyrophosphatase
Smal_2358	0.49	hypothetical protein
Smal_2361	1.58	GntR family transcriptional regulator
Smal_2362	2.11	L-lactate transport
Smal_2365	14.86	AraC family transcriptional regulator
Smal_2366	2.53	hypothetical protein
Smal_2367	5.43	X-Pro dipeptidyl-peptidase domain-containing protein
Smal_2368	-13.52	hypothetical protein
Smal_2369	0.45	major facilitator superfamily protein

Smal_2370	-17.48	LysR family transcriptional regulator
Smal_2371	1.51	beta-glucosidase
Smal_2373	-14.83	AraC family transcriptional regulator
Smal_2376	7.26	DSBA oxidoreductase
Smal_2377	-31.10	cyclase family protein
Smal_2378	5.43	cell division inhibitor SulA
Smal_2379	13.40	DNA repair nucleotidyltransferase/DNA polymerase-like protein
Smal_2380	0.15	error-prone DNA polymerase
Smal_2381	2.71	hypothetical protein
Smal_2387	9.27	ECF subfamily RNA polymerase sigma-24 subunit
Smal_2388	-5.64	anti-FecI sigma factor FecR
Smal_2389	0.23	TonB-dependent receptor
Smal_2390	-19.58	hypothetical protein
Smal_2391	0.47	TonB family protein
Smal_2397	0.39	redoxin domain-containing protein
Smal_2398	4.42	cyclic nucleotide-binding protein
Smal_2401	0.30	hypothetical protein
Smal_2403	0.23	N-acetyltransferase GCN5
Smal_2405	0.30	Pas/Pac sensor-containing methyl-accepting chemotaxis sensory transducer
Smal_2412	20.89	hypothetical protein
Smal_2413	1.81	phage shock protein C, PspC
Smal_2421	2.17	hypothetical protein
Smal_2423	1.63	mechanosensitive ion channel MscS
Smal_2424	-28.09	NADH:flavin oxidoreductase
Smal_2426	1.81	oligoribonuclease
Smal_2427	4.07	zinc-binding CMP/dCMP deaminase
Smal_2429	0.45	NAD(P)H dehydrogenase (quinone)
Smal_2432	1.51	N-acetyltransferase GCN5
Smal_2433	3.62	hypothetical protein
Smal_2449	0.45	hypothetical protein
Smal_2452	3.62	hypothetical protein
Smal_2456	0.18	hypothetical protein
Smal_2457	0.30	hypothetical protein
Smal_2458	2.71	hypothetical protein
Smal_2461	0.18	TonB-dependent siderophore receptor
Smal_2463	-7.75	winged helix family two component transcriptional regulator
Smal_2464	3.62	integral membrane sensor signal transduction histidine kinase
Smal_2466	1.81	RND family efflux transporter MFP subunit

Smal_2467	-8.02	ABC transporter-like protein
Smal_2470	-26.50	hypothetical protein
Smal_2476	0.23	hypothetical protein
Smal_2477	1.81	hypothetical protein
Smal_2479	0.49	hypothetical protein
Smal_2483	0.45	hypothetical protein
Smal_2484	1.81	hypothetical protein
Smal_2485	1.60	hypothetical protein
Smal_2486	-15.63	hypothetical protein
Smal_2488	8.42	carbohydrate-binding CenC domain-containing protein
Smal_2493	12.62	hypothetical protein
Smal_2496	-20.76	phage protein, HK97 gp10 family
Smal_2499	10.04	hypothetical protein
Smal_2501	16.82	peptidase S14 ClpP
Smal_2502	-12.41	phage portal protein, HK97 family
Smal_2503	1.81	terminase
Smal_2505	14.61	HNH endonuclease
Smal_2507	1.81	hypothetical protein
Smal_2508	1.81	hypothetical protein
Smal_2513	-77.14	hypothetical protein
Smal_2514	-14.19	hypothetical protein
Smal_2515	6.62	hypothetical protein
Smal_2517	30.36	hypothetical protein
Smal_2518	2.71	hypothetical protein
Smal_2519	0.45	CI repressor
Smal_2520	2.17	hypothetical protein
Smal_2521	-27.33	hypothetical protein
Smal_2525	-13.63	hypothetical protein
Smal_2526	-20.80	hypothetical protein
Smal_2530	21.96	phage transcriptional regulator AlpA
Smal_2531	1.51	hypothetical protein
Smal_2533	2.26	NAD(P)H dehydrogenase (quinone)
Smal_2534	0.30	Variant SH3 domain-containing protein
Smal_2537	1.81	TonB family protein
Smal_2538	1.88	3-phosphoshikimate 1-carboxyvinyltransferase
Smal_2542	0.34	polyhydroxyalkanoic acid system protein
Smal_2547	2.03	transcriptional regulator, histidine utilization repressor, GntR family
Smal_2553	0.26	beta-lactamase

Smal_2555	0.14	TonB-dependent receptor
Smal_2556	17.91	hypothetical protein
Smal_2557	-13.40	hypothetical protein
Smal_2558	2.11	hypothetical protein
Smal_2559	-9.08	TetR family transcriptional regulator
Smal_2560	8.66	hypothetical protein
Smal_2561	4.17	Agmatine deiminase
Smal_2563	2.26	G-D-S-L family lipolytic protein
Smal_2564	0.18	hypothetical protein
Smal_2566	1.81	hypothetical protein
Smal_2568	-47.60	hypothetical protein
Smal_2573	0.45	beta-lactamase
Smal_2575	-23.07	LysR family transcriptional regulator
Smal_2576	2.03	alcohol dehydrogenase zinc-binding domain-containing protein
Smal_2577	1.51	membrane-bound PQQ-dependent dehydrogenase
Smal_2580	1.81	hypothetical protein
Smal_2593	1.81	hypothetical protein
Smal_2594	4.97	hypothetical protein
Smal_2595	2.58	FMN-binding negative transcriptional regulator
Smal_2597	0.30	3-hydroxyanthranilate 3,4-dioxygenase
Smal_2599	1.96	kynurenine 3-monooxygenase
Smal_2601	2.26	hypothetical protein
Smal_2602	2.49	hypothetical protein
Smal_2604	0.45	inorganic polyphosphate/ATP-NAD kinase
Smal_2619	0.40	hypothetical protein
Smal_2621	1.51	ABC transporter-like protein
Smal_2623	1.61	hypothetical protein
Smal_2624	2.15	hypothetical protein
Smal_2625	0.45	hypothetical protein
Smal_2634	22.27	hypothetical protein
Smal_2636	0.23	TonB-dependent receptor
Smal_2640	14.50	transketolase domain-containing protein
Smal_2644	0.15	hypothetical protein
Smal_2647	10.88	hypothetical protein
Smal_2649	2.21	cold-shock DNA-binding domain-containing protein
Smal_2652	2.11	arsenate reductase
Smal_2656	0.18	ECF subfamily RNA polymerase sigma-24 subunit
Smal_2658	0.41	ABC transporter-like protein

Smal_2660	0.15	glutathione peroxidase
Smal_2665	2.71	hypothetical protein
Smal_2677	1.58	RluA family pseudouridine synthase
Smal_2679	42.29	endonuclease/exonuclease/phosphatase
Smal_2680	1.75	coagulation factor 5/8 type domain-containing protein
Smal_2681	0.45	monosaccharide-transporting ATPase
Smal_2683	0.30	family 1 extracellular solute-binding protein
Smal_2684	3.44	hypothetical protein
Smal_2685	1.92	TonB-dependent receptor
Smal_2686	1.81	LacI family transcriptional regulator
Smal_2687	1.81	BolA family protein
Smal_2688	3.62	hypothetical protein
Smal_2690	0.50	segregation and condensation protein B
Smal_2694	0.18	hypothetical protein
Smal_2695	-53.45	hypothetical protein
Smal_2696	0.26	hypothetical protein
Smal_2697	1.81	nitrilase/cyanide hydratase and apolipoprotein N-acyltransferase
Smal_2698	2.17	putative aminotransferase
Smal_2701	1.81	heme exporter protein CcmC
Smal_2702	1.81	heme exporter protein CcmD
Smal_2706	2.11	cytochrome C biogenesis protein
Smal_2707	2.41	hypothetical protein
Smal_2709	-18.00	hypothetical protein
Smal_2714	1.81	cyd operon protein YbgT
Smal_2716	0.45	HmsH protein
Smal_2717	-5.54	polysaccharide deacetylase
Smal_2718	3.76	N-glycosyltransferase
Smal_2719	0.45	hypothetical protein
Smal_2721	0.23	gamma-glutamyl kinase
Smal_2723	0.41	argininosuccinate lyase
Smal_2725	0.50	amino-acid acetyltransferase
Smal_2733	0.30	major facilitator superfamily protein
Smal_2750	3.16	acetoacetyl-CoA reductase
Smal_2753	1.63	hypothetical protein
Smal_2758	1.58	iron-sulfur cluster binding protein
Smal_2759	1.81	exodeoxyribonuclease VII large subunit
Smal_2760	2.71	peptidase M48 Ste24p
Smal_2763	-25.25	hypothetical protein

Smal_2765	-8.85	TetR family transcriptional regulator
Smal_2766	0.45	Xylose isomerase domain-containing protein
Smal_2769	54.57	hypothetical protein
Smal_2771	6.20	short chain dehydrogenase
Smal_2772	10.03	AraC family transcriptional regulator
Smal_2773	-5.09	alcohol dehydrogenase zinc-binding domain-containing protein
Smal_2775	1.81	lipocalin family protein
Smal_2780	0.45	hypothetical protein
Smal_2783	46.74	hypothetical protein
Smal_2784	4.52	hypothetical protein
Smal_2787	2.11	small multidrug resistance protein
Smal_2789	0.34	lysine exporter protein LysE/YggA
Smal_2790	0.45	ATP-dependent Clp protease proteolytic subunit
Smal_2792	2.11	ArsR family transcriptional regulator
Smal_2793	2.71	aspartate racemase
Smal_2794	59.75	hypothetical protein
Smal_2795	-10.26	hypothetical protein
Smal_2796	-59.15	hypothetical protein
Smal_2797	0.36	N-acetyltransferase GCN5
Smal_2798	1.58	MerR family transcriptional regulator
Smal_2807	1.81	hypothetical protein
Smal_2810	-5.05	hypothetical protein
Smal_2823	0.50	NADH dehydrogenase subunit I
Smal_2827	1.63	NADH dehydrogenase subunit E
Smal_2844	-5.89	LysR family transcriptional regulator
Smal_2846	-7.38	signal recognition particle-docking protein FtsY
Smal_2849	2.26	hypothetical protein
Smal_2852	52.16	Glyoxalase/bleomycin resistance protein/dioxygenase
Smal_2853	1.56	hypothetical protein
Smal_2858	0.50	electron transport protein SCO1/SenC
Smal_2864	-12.49	putative putative transmembrane protein
Smal_2868	3.62	hypothetical protein
Smal_2878	1.81	amine oxidase
Smal_2880	15.63	methylated-DNA--protein-cysteine methyltransferase
Smal_2881	0.45	hypothetical protein
Smal_2883	2.11	pyridine nucleotide-disulfide oxidoreductase dimerisation protein
Smal_2885	0.15	hypothetical protein
Smal_2888	1.81	NLP/P60 protein

Smal_2891	17.42	hypothetical protein
Smal_2892	1.51	tRNA(Ile)-lysidine synthetase
Smal_2893	1.51	exodeoxyribonuclease VII small subunit
Smal_2894	1.81	polyprenyl synthetase
Smal_2903	1.63	TonB family protein
Smal_2904	1.52	TonB-dependent receptor
Smal_2905	2.41	arginine/ornithine antiporter
Smal_2906	2.03	xylanase
Smal_2909	0.15	nicotinic acid mononucleotide adenylyltransferase
Smal_2913	-17.52	hypothetical protein
Smal_2917	3.32	hypothetical protein
Smal_2919	5.43	hypothetical protein
Smal_2920	20.73	hypothetical protein
Smal_2921	0.30	hypothetical protein
Smal_2924	0.18	Glyoxalase/bleomycin resistance protein/dioxygenase
Smal_2925	2.03	hypothetical protein
Smal_2927	2.07	HAD-superfamily hydrolase
Smal_2928	1.81	ferredoxin
Smal_2929	2.11	endoribonuclease L-PSP
Smal_2931	0.40	virulence factor family protein
Smal_2933	50.22	hypothetical protein
Smal_2934	2.71	hypothetical protein
Smal_2935	2.71	hypothetical protein
Smal_2936	1.63	alkylphosphonate utilization operon protein PhnA
Smal_2937	1.58	heat shock protein
Smal_2944	0.36	hypothetical protein
Smal_2947	1.94	hypothetical protein
Smal_2949	3.16	hypothetical protein
Smal_2950	0.39	RNA-binding S4 domain-containing protein
Smal_2956	1.85	outer membrane protein
Smal_2961	-10.61	DEAD/DEAH box helicase
Smal_2962	0.45	metallophosphoesterase
Smal_2964	0.36	monooxygenase FAD-binding
Smal_2966	2.98	5'-methylthioadenosine phosphorylase
Smal_2971	1.68	23S rRNA 5-methyluridine methyltransferase
Smal_2974	1.57	GTP-binding protein Era
Smal_2990	-40.26	Glyoxalase/bleomycin resistance protein/dioxygenase
Smal_2992	5.43	winged helix family two component transcriptional regulator

Smal_2994	-13.61	hypothetical protein
Smal_2996	1.85	peptidyl-dipeptidase A
Smal_2997	0.30	hypothetical protein
Smal_2998	-23.30	FAD-binding 9 siderophore-interacting domain-containing protein
Smal_2999	1.99	Bcr/CflA subfamily drug resistance transporter
Smal_3003	2.11	AraC family transcriptional regulator
Smal_3006	2.11	hypothetical protein
Smal_3007	1.58	methyl-accepting chemotaxis sensory transducer
Smal_3009	1.92	hypothetical protein
Smal_3010	0.18	ErfK/YbiS/YcfS/YnhG family protein
Smal_3012	-7.81	hypothetical protein
Smal_3014	0.30	hypothetical protein
Smal_3017	7.23	YhdH/YhfP family quinone oxidoreductase
Smal_3022	1.58	penicillin-binding protein 1C
Smal_3027	2.03	putative addiction module antidote protein
Smal_3032	2.26	DoxX family protein
Smal_3038	3.62	hypothetical protein
Smal_3042	0.47	integral membrane sensor hybrid histidine kinase
Smal_3043	1.81	hypothetical protein
Smal_3044	0.30	cytochrome B561
Smal_3046	62.38	glutaredoxin
Smal_3047	1.73	L-serine dehydratase 1
Smal_3048	8.14	alpha/beta hydrolase fold domain-containing protein
Smal_3049	0.18	Homoserine dehydrogenase
Smal_3050	0.36	cystathionine gamma-synthase
Smal_3051	0.30	homoserine O-acetyltransferase
Smal_3052	-5.85	peptidase M23
Smal_3054	3.32	hemolysin III family channel protein
Smal_3055	0.45	AsmA family protein
Smal_3056	0.30	putative signal transduction protein
Smal_3059	1.74	Hsp33 protein
Smal_3066	-44.74	hypothetical protein
Smal_3067	3.62	hypothetical protein
Smal_3069	18.68	short-chain dehydrogenase/reductase SDR
Smal_3070	0.45	LysR family transcriptional regulator
Smal_3074	4.52	hypothetical protein
Smal_3076	4.52	hypothetical protein
Smal_3077	0.45	hypothetical protein

Smal_3083	1.51	CheW protein
Smal_3084	1.81	hypothetical protein
Smal_3085	1.50	CheA signal transduction histidine kinase
Smal_3087	1.55	CheW protein
Smal_3089	2.35	response regulator receiver protein
Smal_3090	2.03	glutathione synthetase
Smal_3095	1.51	penicillin-binding protein 1B
Smal_3096	1.55	family 2 glycosyl transferase
Smal_3097	0.45	NAD-dependent epimerase/dehydratase
Smal_3098	2.71	hypothetical protein
Smal_3107	46.10	N-acetyltransferase GCN5
Smal_3109	4.52	abortive infection protein
Smal_3110	22.21	response regulator receiver protein
Smal_3112	-18.60	putative esterase
Smal_3113	1.58	hypothetical protein
Smal_3114	1.81	hypothetical protein
Smal_3124	2.51	Holliday junction DNA helicase RuvB
Smal_3125	0.39	K potassium transporter
Smal_3133	19.06	hypothetical protein
Smal_3134	4.97	hypothetical protein
Smal_3135	4.52	sodium/hydrogen exchanger
Smal_3136	2.71	beta-lactamase
Smal_3137	1.81	LysR family transcriptional regulator
Smal_3141	3.62	O-acetylhomoserine/O-acetylserine sulfhydrylase
Smal_3142	0.18	winged helix family two component transcriptional regulator
Smal_3143	1.81	integral membrane sensor signal transduction histidine kinase
Smal_3144	2.35	MltA-interacting MipA family protein
Smal_3149	4.07	thiamine pyrophosphate domain-containing TPP-binding protein
Smal_3150	1.68	hypothetical protein
Smal_3151	1.51	hypothetical protein
Smal_3153	2.07	HAD-superfamily hydrolase
Smal_3155	2.05	alpha,alpha-trehalose-phosphate synthase
Smal_3156	1.81	putative thiol-disulfide oxidoreductase DCC
Smal_3157	2.11	hypothetical protein
Smal_3159	4.07	RluA family pseudouridine synthase
Smal_3161	3.16	hypothetical protein
Smal_3169	1.51	group 1 glycosyl transferase
Smal_3176	1.51	fimbrial protein pilin

Smal_3180	1.51	integral membrane sensor signal transduction histidine kinase
Smal_3184	0.18	alpha-L-glutamate ligase
Smal_3187	0.49	hypothetical protein
Smal_3189	70.81	sulfur carrier protein ThiS
Smal_3192	1.58	TrkA-C domain-containing protein
Smal_3194	0.45	hypothetical protein
Smal_3197	1.81	peptidase M28
Smal_3198	0.45	amino acid carrier protein
Smal_3201	1.81	amino acid carrier protein
Smal_3203	2.41	acriflavin resistance protein
Smal_3204	-4.72	RND family efflux transporter MFP subunit
Smal_3206	0.30	hypothetical protein
Smal_3209	0.25	transcriptional regulator CysB-like protein
Smal_3215	2.86	HAD-superfamily hydrolase
Smal_3221	2.11	S1/P1 nuclease
Smal_3224	1.58	TonB family protein
Smal_3226	2.71	flavin reductase domain-containing protein
Smal_3232	2.49	von Willebrand factor type A
Smal_3233	1.81	hypothetical protein
Smal_3234	0.30	hypothetical protein
Smal_3236	2.00	type IV pilus secretin PilQ
Smal_3241	1.81	penicillin-binding protein
Smal_3242	3.62	hypothetical protein
Smal_3244	-27.88	hypothetical protein
Smal_3246	0.45	putative fimbrial protein
Smal_3248	0.45	outermembrane fimbrial usher protein
Smal_3249	-10.44	CS1 type fimbrial major subunit
Smal_3252	-20.26	AraC family transcriptional regulator
Smal_3257	1.51	endoribonuclease L-PSP
Smal_3265	1.72	L-seryl-tRNA(Sec) selenium transferase
Smal_3272	2.61	putative deoxyribonucleotide triphosphate pyrophosphatase
Smal_3274	1.74	hypothetical protein
Smal_3276	1.81	proline dipeptidase
Smal_3279	0.18	hypothetical protein
Smal_3284	1.63	hypothetical protein
Smal_3290	23.52	HAD-superfamily hydrolase
Smal_3291	1.55	acetylornithine transaminase protein
Smal_3292	4.52	Ion transport protein

Smal_3295	2.26	short chain dehydrogenase
Smal_3298	3.16	serine/threonine protein kinase
Smal_3299	1.81	PA-phosphatase-like phosphoesterase
Smal_3305	5.43	hypothetical protein
Smal_3306	1.67	hypothetical protein
Smal_3309	-24.57	TonB system transport protein ExbD
Smal_3310	1.81	tonB-system energizer ExbB
Smal_3311	-11.18	hypothetical protein
Smal_3312	-8.89	heme oxygenase-like protein
Smal_3313	36.73	hypothetical protein
Smal_3314	0.23	TonB-dependent receptor
Smal_3315	1.81	anti-FecI sigma factor FecR
Smal_3316	0.30	ECF subfamily RNA polymerase sigma-24 subunit
Smal_3318	1.91	inorganic pyrophosphatase
Smal_3322	0.40	AraC family transcriptional regulator
Smal_3327	5.33	gamma-glutamyltransferase
Smal_3328	1.58	ketol-acid reductoisomerase
Smal_3331	1.81	threonine dehydratase
Smal_3332	2.17	2-isopropylmalate synthase
Smal_3333	12.21	type 11 methyltransferase
Smal_3335	1.81	3-isopropylmalate dehydratase small subunit
Smal_3336	1.81	3-isopropylmalate dehydrogenase
Smal_3337	12.21	short-chain dehydrogenase/reductase SDR
Smal_3341	1.52	TetR family transcriptional regulator
Smal_3342	1.53	protein-L-isoaspartate(D-aspartate) O-methyltransferase
Smal_3344	0.15	hypothetical protein
Smal_3348	0.45	group 1 glycosyl transferase
Smal_3349	119.81	hypothetical protein
Smal_3350	2.29	alpha-1,2-mannosidase
Smal_3351	2.07	hypothetical protein
Smal_3355	2.07	PAS/PAC sensor hybrid histidine kinase
Smal_3356	0.23	two component LuxR family transcriptional regulator
Smal_3357	1.81	family 1 extracellular solute-binding protein
Smal_3358	0.23	integral membrane sensor signal transduction histidine kinase
Smal_3359	0.30	winged helix family two component transcriptional regulator
Smal_3363	-6.82	TonB-dependent receptor
Smal_3375	2.71	DSBA oxidoreductase
Smal_3378	0.30	NodT family RND efflux system outer membrane lipoprotein

Smal_3380	0.45	major facilitator superfamily protein
Smal_3381	0.41	LysR family transcriptional regulator
Smal_3383	1.81	hypothetical protein
Smal_3384	6.53	diguanylate cyclase
Smal_3389	22.53	hypothetical protein
Smal_3391	1.51	NADH:flavin oxidoreductase
Smal_3393	5.43	hypothetical protein
Smal_3399	1.81	hypothetical protein
Smal_3400	1.81	AraC family transcriptional regulator
Smal_3403	1.66	cytochrome c class I
Smal_3407	-5.97	hypothetical protein
Smal_3408	9.79	AsnC family transcriptional regulator
Smal_3411	0.43	TonB-dependent receptor
Smal_3415	2.33	multifunctional tRNA nucleotidyl transferase
Smal_3417	0.30	hypothetical protein
Smal_3418	-35.34	peptidase S41
Smal_3422	1.66	glutamine--fructose-6-phosphate transaminase
Smal_3425	3.01	glucokinase
Smal_3426	1.81	TonB-dependent receptor
Smal_3427	2.71	beta-N-acetylhexosaminidase
Smal_3432	2.26	20S proteasome subunits A and B
Smal_3433	1.53	response regulator receiver sensor hybrid histidine kinase
Smal_3438	1.85	6-phosphogluconate dehydrogenase
Smal_3440	4.97	hypothetical protein
Smal_3445	0.48	hypothetical protein
Smal_3447	1.56	rare lipoprotein A
Smal_3449	-19.35	rod shape-determining protein RodA
Smal_3451	3.32	penicillin-binding protein 2
Smal_3458	3.26	hypothetical protein
Smal_3462	0.45	hypothetical protein
Smal_3466	1.55	NodT family RND efflux system outer membrane lipoprotein
Smal_3468	1.59	RND family efflux transporter MFP subunit
Smal_3475	1.68	hypothetical protein
Smal_3476	1.95	diaminopimelate epimerase
Smal_3477	0.30	hypothetical protein
Smal_3482	0.18	hypothetical protein
Smal_3484	1.81	binding-protein-dependent transport system inner membrane protein
Smal_3487	2.17	putative nucleotide-binding protein

Smal_3490	1.56	aldehyde dehydrogenase
Smal_3491	2.71	hypothetical protein
Smal_3492	2.49	short-chain dehydrogenase/reductase SDR
Smal_3494	2.71	hypothetical protein
Smal_3496	1.77	lactoylglutathione lyase
Smal_3501	0.45	ABC transporter-like protein
Smal_3502	0.30	hypothetical protein
Smal_3504	12.23	hypothetical protein
Smal_3505	1.81	two component sigma54 specific Fis family transcriptional regulator
Smal_3506	1.51	integral membrane sensor signal transduction histidine kinase
Smal_3509	5.43	GtrA family protein
Smal_3515	1.67	F0F1 ATP synthase subunit B
Smal_3522	0.30	hypothetical protein
Smal_3524	0.30	glycerol-3-phosphate dehydrogenase
Smal_3525	-17.41	glycerol kinase
Smal_3528	0.13	hypothetical protein
Smal_3532	2.26	histidine kinase internal region
Smal_3538	0.34	NAD-dependent epimerase/dehydratase
Smal_3541	1.81	hypothetical protein
Smal_3542	0.30	peptidase M24
Smal_3545	-3.55	EmrB/QacA subfamily drug resistance transporter
Smal_3546	0.23	TetR family transcriptional regulator
Smal_3547	1.81	Glyoxalase/bleomycin resistance protein/dioxygenase
Smal_3548	-52.20	hypothetical protein
Smal_3549	1.81	outer membrane autotransporter barrel domain-containing protein
Smal_3551	0.18	LysR family transcriptional regulator
Smal_3552	0.30	major facilitator superfamily protein
Smal_3554	2.26	peptidoglycan glycosyltransferase
Smal_3555	0.45	hypothetical protein
Smal_3556	0.36	TonB-dependent receptor
Smal_3557	-10.22	hypothetical protein
Smal_3559	3.01	AraC family transcriptional regulator
Smal_3560	2.11	glutathione S-transferase domain-containing protein
Smal_3561	0.45	hypothetical protein
Smal_3563	0.45	DoxX family protein
Smal_3564	3.32	hypothetical protein
Smal_3569	0.18	ATPase AAA
Smal_3570	1.63	hypothetical protein

Smal_3571	1.60	hypothetical protein
Smal_3573	1.99	D-tyrosyl-tRNA(Tyr) deacylase
Smal_3575	1.59	GTP cyclohydrolase
Smal_3576	2.11	membrane-flanked domain-containing protein
Smal_3578	0.50	CDP-glycerol:poly(glycerophosphate) glycerophosphotransferase
Smal_3579	0.23	family 2 glycosyl transferase
Smal_3582	0.42	glycosyl transferase family protein
Smal_3586	2.26	peptidoglycan-binding LysM
Smal_3589	1.97	fimbrial protein pilin
Smal_3592	2.41	SUA5/yciO/yrdC domain
Smal_3593	4.52	hypothetical protein
Smal_3596	-24.62	hypothetical protein
Smal_3600	18.81	hypothetical protein
Smal_3605	-57.74	hypothetical protein
Smal_3606	10.95	hypothetical protein
Smal_3609	1.51	hypothetical protein
Smal_3610	4.52	RND family efflux transporter MFP subunit
Smal_3615	2.49	hypothetical protein
Smal_3618	1.66	MarR family transcriptional regulator
Smal_3619	0.36	integral membrane sensor signal transduction histidine kinase
Smal_3620	-14.77	winged helix family two component transcriptional regulator
Smal_3621	1.81	hypothetical protein
Smal_3623	22.30	glutathione S-transferase domain-containing protein
Smal_3624	0.15	LysR family transcriptional regulator
Smal_3625	7.69	hypothetical protein
Smal_3628	6.33	endonuclease I
Smal_3629	1.51	CutA1 divalent ion tolerance protein
Smal_3630	0.41	C-type cytochrome biogenesis protein
Smal_3631	1.51	alkyl hydroperoxide reductase
Smal_3632	0.45	flavodoxin/nitric oxide synthase
Smal_3634	-24.89	N-hydroxyarylamine O-acetyltransferase
Smal_3635	-14.07	Glyoxalase/bleomycin resistance protein/dioxygenase
Smal_3636	3.62	family 3 extracellular solute-binding protein
Smal_3637	1.81	LysR family transcriptional regulator
Smal_3639	2.11	hypothetical protein
Smal_3641	7.23	TetR family transcriptional regulator
Smal_3644	-12.58	hypothetical protein
Smal_3647	2.41	hypothetical protein

Smal_3648	1.56	acetyl-CoA carboxylase biotin carboxyl carrier protein subunit
Smal_3651	2.16	hypothetical protein
Smal_3653	2.26	hypothetical protein
Smal_3654	0.45	DNA-binding protein Fis
Smal_3655	0.45	phosphatidate cytidylyltransferase
Smal_3656	1.81	phospholipid/glycerol acyltransferase
Smal_3657	64.81	hypothetical protein
Smal_3658	2.17	putative dual specificity phosphatase
Smal_3659	0.45	hypothetical protein
Smal_3661	-8.38	hypothetical protein
Smal_3663	2.94	hypothetical protein
Smal_3667	0.45	CopY family transcriptional repressor
Smal_3675	2.33	thioredoxin
Smal_3676	1.55	transcription termination factor Rho
Smal_3679	0.18	bifunctional isocitrate dehydrogenase kinase/phosphatase protein
Smal_3680	39.45	hypothetical protein
Smal_3682	-9.53	hypothetical protein
Smal_3683	0.36	hypothetical protein
Smal_3685	1.68	hypothetical protein
Smal_3687	2.20	7-cyano-7-deazaguanine reductase
Smal_3688	1.68	amidohydrolase
Smal_3691	0.49	acriflavin resistance protein
Smal_3692	0.26	TonB-dependent siderophore receptor
Smal_3693	0.50	YccS/YhfK family integral membrane protein
Smal_3694	-13.15	short-chain dehydrogenase/reductase SDR
Smal_3707	1.51	bacterioferritin
Smal_3708	0.23	BFD (2Fe-2S)-binding domain-containing protein
Smal_3712	4.07	ubiquinone biosynthesis protein
Smal_3713	0.30	small multidrug resistance protein
Smal_3715	1.51	cAMP-regulatory protein
Smal_3716	0.18	hypothetical protein
Smal_3717	2.26	haloacid dehalogenase domain-containing protein hydrolase
Smal_3723	2.20	lipid kinase
Smal_3726	1.81	heat shock protein DnaJ domain-containing protein
Smal_3727	2.26	phosphoribosylaminoimidazole-succinocarboxamide synthase
Smal_3730	2.45	putative monovalent cation/H+ antiporter subunit C
Smal_3732	0.45	putative monovalent cation/H+ antiporter subunit E
Smal_3734	1.51	putative monovalent cation/H+ antiporter subunit G

Smal_3735	0.30	sodium/calcium exchanger membrane protein
Smal_3741	0.45	hypothetical protein
Smal_3743	3.01	putative thioredoxin
Smal_3749	1.53	branched-chain alpha-keto acid dehydrogenase subunit E2
Smal_3750	0.30	hypothetical protein
Smal_3752	-7.38	helix-turn-helix type 11 domain-containing protein
Smal_3754	0.36	diguanylate cyclase
Smal_3756	1.51	ABC-1 domain-containing protein
Smal_3757	1.81	phosphoglycerol transferase I
Smal_3759	0.15	hypothetical protein
Smal_3761	0.18	FAD dependent oxidoreductase
Smal_3763	0.30	hypothetical protein
Smal_3767	0.45	hypothetical protein
Smal_3769	1.54	spermidine synthase
Smal_3771	0.30	Mn2+-dependent serine/threonine protein kinase
Smal_3774	3.16	rifampin ADP-ribosyl transferase
Smal_3777	0.15	hypothetical protein
Smal_3779	66.30	hypothetical protein
Smal_3782	0.30	Mg chelatase subunit ChlI
Smal_3785	2.26	sugar phosphatase
Smal_3787	1.58	L-sorbosone dehydrogenase
Smal_3788	2.71	hypothetical protein
Smal_3789	2.03	peptidase S8/S53 subtilisin kexin sedolisin
Smal_3791	-6.57	electron transport protein SCO1/SenC
Smal_3793	-16.44	cytochrome o ubiquinol oxidase subunit III
Smal_3797	-20.08	hypothetical protein
Smal_3798	7.88	hypothetical protein
Smal_3799	4.52	GntR family transcriptional regulator
Smal_3800	0.23	class V aminotransferase
Smal_3801	0.45	TonB-dependent receptor
Smal_3802	1.51	hypothetical protein
Smal_3804	0.45	hypothetical protein
Smal_3806	1.81	hypothetical protein
Smal_3811	34.79	hypothetical protein
Smal_3814	0.34	glycoside hydrolase family protein
Smal_3816	2.71	ROK family protein
Smal_3817	-7.83	D-tagatose-bisphosphate aldolase non-catalytic subunit
Smal_3818	1.81	DNA-binding transcriptional regulator AgaR

Smal_3820	0.30	general substrate transporter
Smal_3821	1.81	hypothetical protein
Smal_3823	-7.51	competence protein F
Smal_3824	0.30	hypothetical protein
Smal_3827	3.62	biotin synthase
Smal_3828	4.52	8-amino-7-oxononanoate synthase
Smal_3829	1.51	bioH protein
Smal_3835	1.58	tRNA uridine 5-carboxymethylaminomethyl modification protein GidA
Smal_3838	19.73	RND family efflux transporter MFP subunit
Smal_3839	-11.26	integral membrane sensor signal transduction histidine kinase
Smal_3840	0.23	winged helix family two component transcriptional regulator
Smal_3841	1.51	hypothetical protein
Smal_3843	2.11	RDD domain-containing protein
Smal_3847	0.30	ATPase
Smal_3848	2.26	hypothetical protein
Smal_3850	1.61	hypothetical protein
Smal_3851	2.41	dihydroxy-acid dehydratase
Smal_3855	2.71	hypothetical protein
Smal_3856	1.89	shikimate 5-dehydrogenase
Smal_3857	2.56	WD40 domain-containing protein
Smal_3860	1.94	hypothetical protein
Smal_3861	2.26	hypothetical protein
Smal_3862	0.30	hypothetical protein
Smal_3864	1.81	hypothetical protein
Smal_3866	2.26	glutathione S-transferase domain-containing protein
Smal_3867	1.81	hypothetical protein
Smal_3868	2.94	TonB family protein
Smal_3869	37.17	hypothetical protein
Smal_3870	-13.10	AraC family transcriptional regulator
Smal_3871	-7.35	hypothetical protein
Smal_3877	0.18	chitin-binding protein
Smal_3878	2.71	chitin-binding protein
Smal_3882	0.39	hypothetical protein
Smal_3884	2.71	NAD-dependent epimerase/dehydratase
Smal_3885	1.81	TatD-related deoxyribonuclease
Smal_3886	3.16	ATP-dependent helicase HrpB
Smal_3888	2.26	pseudouridine synthase
Smal_3890	1.58	putative methyltransferase

Smal_3894	4.07	FAD-dependent pyridine nucleotide-disulfide oxidoreductase
Smal_3897	1.51	LysR family transcriptional regulator
Smal_3900	13.35	alpha/beta hydrolase fold domain-containing protein
Smal_3901	-13.74	hypothetical protein
Smal_3907	1.88	hypothetical protein
Smal_3912	-18.74	hypothetical protein
Smal_3916	1.58	thioesterase superfamily protein
Smal_3917	0.50	Histidine ammonia-lyase
Smal_3918	10.85	lipid A biosynthesis acyltransferase
Smal_3922	1.51	acyl carrier protein
Smal_3925	0.45	hypothetical protein
Smal_3926	3.62	cytochrome d ubiquinol oxidase subunit II
Smal_3927	2.26	cytochrome bd ubiquinol oxidase subunit I
Smal_3928	0.39	hypothetical protein
Smal_3929	0.45	hypothetical protein
Smal_3931	3.62	ECF subfamily RNA polymerase sigma-24 subunit
Smal_3932	28.70	hypothetical protein
Smal_3935	-27.96	hypothetical protein
Smal_3936	4.21	hypothetical protein
Smal_3937	3.16	hypothetical protein
Smal_3940	3.62	hypothetical protein
Smal_3949	1.58	phospholipase D/transphosphatidylase
Smal_3952	5.43	DNA polymerase III subunit epsilon
Smal_3956	1.66	outer membrane efflux protein
Smal_3957	1.71	RND family efflux transporter MFP subunit
Smal_3960	-11.06	integrase catalytic subunit
Smal_3964	1.54	hypothetical protein
Smal_3965	2.28	parB-like partition protein
Smal_3967	1.81	16S rRNA methyltransferase GidB
Smal_3968	3.62	4'-phosphopantetheinyl transferase
Smal_3969	2.89	transglycosylase-associated protein
Smal_3970	2.26	exodeoxyribonuclease III
Smal_3971	2.03	aldehyde dehydrogenase
Smal_3973	1.99	hypothetical protein
Smal_3974	1.93	acetyl-CoA synthetase
Smal_3975	1.69	two component LuxR family transcriptional regulator
Smal_3977	0.30	hypothetical protein
Smal_3978	1.55	multi-sensor hybrid histidine kinase

Smal_3981	1.51	surface antigen (D15)
Smal_3982	1.58	glycyl-tRNA synthetase subunit beta
Smal_3985	1.51	glutamine amidotransferase
Smal_3986	2.35	hypothetical protein
Smal_3987	0.27	Sec-independent protein translocase subunit TatC
Smal_3992	3.92	alpha/beta hydrolase fold domain-containing protein
Smal_3993	0.45	Pas/Pac sensor-containing methyl-accepting chemotaxis sensory transducer
Smal_3995	0.35	hypothetical protein
Smal_3999	0.30	hypothetical protein
Smal_4003	1.51	hypothetical protein
Smal_4004	1.91	hypothetical protein
Smal_4007	1.70	MarR family transcriptional regulator
Smal_4008	0.36	hypothetical protein
Smal_4011	0.41	fusaric acid resistance protein
Smal_4012	0.45	hypothetical protein
Smal_4013	1.51	exodeoxyribonuclease V subunit alpha
Smal_4014	2.08	exodeoxyribonuclease V subunit beta
Smal_4020	2.44	hypothetical protein
Smal_4023	-52.46	hypothetical protein
Smal_4025	-25.88	glutathione S-transferase domain-containing protein
Smal_4029	0.30	hypothetical protein
Smal_4030	2.71	hypothetical protein
Smal_4037	2.17	rhomboid family protein
Smal_4038	1.63	glycerophosphoryl diester phosphodiesterase
Smal_4040	0.36	putative phage-like protein
Smal_4041	2.26	tRNA modification GTPase TrmE
Smal_R0001	-69.60	tRNA-Thr
Smal_R0011	41.41	5S ribosomal RNA
Smal_R0020	2.26	tRNA-Arg1
Smal_R0023	0.49	tRNA-Lys2
Smal_R0024	-62.14	tRNA-Leu1
Smal_R0030	104.90	tRNA-Ser1
Smal_R0031	41.41	SRP RNA; RNA component of signal recognition particle
Smal_R0033	63.79	tRNA-Val2
Smal_R0035	-60.70	tRNA-Leu7
Smal_R0036	-60.70	tRNA-Leu6
Smal_R0037	168.58	tRNA-Leu5
Smal_R0042	-69.60	tRNA-Gly3

Smal_R0044	-69.60	tRNA-Gly5
Smal_R0045	-71.51	tRNA-Cys1
Smal_R0046	0.18	tRNA-Leu4
Smal_R0047	3.62	tRNA-Ser2
Smal_R0051	-70.54	tRNA-Glu4
Smal_R0054	2.56	tRNA-Ala3
Smal_R0061	-58.65	tRNA-Ser3
Smal_R0063	62.11	tRNA-Pro3
Smal_R0064	62.94	tRNA-Ala6
Smal_R0073	1.51	tRNA-Gly6
Smal_R0074	4.52	tRNA-SeC1
Smal_R0078	2.41	tRNA-Gln2
Smal_R0080	0.38	tRNA-Arg4
Smal_R0081	82.81	5S ribosomal RNA
